# Codon usage bias in chloroplast genes implicate adaptive evolution of four ginger species

**DOI:** 10.3389/fpls.2023.1304264

**Published:** 2023-12-15

**Authors:** Qian Yang, Cheng Xin, Qing-Song Xiao, Ya-Ting Lin, Li Li, Jian-Li Zhao

**Affiliations:** ^1^ Ministry of Education Key Laboratory for Transboundary Ecosecurity of Southwest China, Yunnan Key Laboratory of Plant Reproductive Adaptation and Evolutionary Ecology and Centre for Invasion Biology, Institute of Biodiversity, School of Ecology and Environmental Science, Yunnan University, Kunming, Yunnan, China; ^2^ School of Life Sciences, Zhengzhou University, Zhengzhou, China

**Keywords:** codon usage bias, chloroplast genomes, adaptive evolution, natural selection, mutation pressure

## Abstract

Codon usage bias (CUB) refers to different codons exhibiting varying frequencies of usage in the genome. Studying CUB is crucial for understanding genome structure, function, and evolutionary processes. Herein, we investigated the codon usage patterns and influencing factors of protein-coding genes in the chloroplast genomes of four sister genera (monophyletic *Roscoea* and *Cautleya*, and monophyletic *Pommereschea* and *Rhynchanthus*) from the Zingiberaceae family with contrasting habitats in southwestern China. These genera exhibit distinct habitats, providing a unique opportunity to explore the adaptive evolution of codon usage. We conducted a comprehensive analysis of nucleotide composition and codon usage on protein-coding genes in the chloroplast genomes. The study focused on understanding the relationship between codon usage and environmental adaptation, with a particular emphasis on genes associated with photosynthesis. Nucleotide composition analysis revealed that the overall G/C content of the coding genes was ˂ 48%, indicating an enrichment of A/T bases. Additionally, synonymous and optimal codons were biased toward ending with A/U bases. Natural selection is the primary factor influencing CUB characteristics, particularly photosynthesis-associated genes. We observed differential gene expressions related to light adaptation among sister genera inhabiting different environments. Certain codons were favored under specific conditions, possibly contributing to gene expression regulation in particular environments. This study provides insights into the adaptive evolution of these sister genera by analyzing CUB and offers theoretical assistance for understanding gene expression and regulation. In addition, the data support the relationship between RNA editing and CUB, and the findings shed light on potential research directions for investigating adaptive evolution.

## 1 Introduction

Plant codons are three-base (nucleotide) units in DNA sequences. Codons act as links between nucleic acids (DNA and RNA) and proteins, providing instructions for translating the genetic information in DNA from RNA into proteins, and play a crucial role in the process of genetic information transfer (Radwan et al., 2010). Plants contain 64 codons, with each codon corresponding to an amino acid or termination signal. Codon usage bias (CUB) refers to the unequal use of synonymous codons during gene transcription (Jia and Xue, 2009; Wei et al., 2014). This phenomenon is widely observed in numerous organisms in a non-random manner and believed to reflect certain evolutionary events in cellular and environmental adaptations (Botzman and Margalit, 2011). CUB can be influenced by various factors, including genetic and environmental factors, as well as evolutionary pressures (Sharp et al., 1988; Shackelton et al., 2006; Tao et al., 2009; Pandey et al., 2020). Current research suggests that CUB is primarily the result of a balance between mutational bias and natural selection pressure (Gu et al., 2004; Tyagi et al., 2023). Therefore, studying CUB is essential for understanding the functionality and evolutionary mechanisms of plant genomes and can contribute to understanding the evolutionary and environmental adaptations of related species.

The chloroplast (cp) genome of typical angiosperms comprises a circular, double-stranded DNA molecule ranging from 120 to 160 kb (Yang et al., 2020). It contains approximately 130 genes distributed within two identical inverted repeat regions, referred to as the large and small single-copy regions (Xin and Yang, 2020; Yang et al., 2021). Due to its stable structure and uniparental inheritance, the cp genome has been used for comparative analyses to understand the evolution of closely related species. CUB plays a significant role in gene expression, therefore, research on codons in the cp genome is highly important (Song et al., 2017; Yang et al., 2018). By studying the codon usage patterns of plants from different habitats, researchers can identify specific codons or amino acids that are preferred or avoided in response to environmental factors, such as temperature, light and nutrient availabilities, and water stress. Analyzing codon usage patterns in stress-related genes, of which CUB may be associated, can provide deeper insights into how plants respond to biotic and abiotic stresses, such as drought, salinity, pathogens, or extreme temperatures. For example, studies have shown correlations between gene expression levels and synonymous codon usage in rice under biotic or abiotic stresses, such as drought stress (Chamani Mohasses et al., 2020; Tyagi et al., 2023). However, from the perspective of codon usage patterns, research on the adaptive evolution of plants to different habitats is relatively limited at present.

Recent research has revealed the potential relationship between plant RNA editing and CUB. RNA editing is a post-transcriptional modification process that alters the coding sequence of mRNA, consequently influencing protein translation and function (Wang et al., 2015; Wang et al., 2016). This phenomenon plays a critical role in gene expression regulation and adaptation in plants (Ichinose and Sugita, 2016). Similarly, CUB involves the preferential usage of certain synonymous codons over others and can affect gene regulation. Although the relationship between RNA editing and CUB is not direct, both processes may contribute to the overall regulation of plant genes. Theoretically, CUB could influence RNA editing in specific regions of the genome, and the different frequencies of codon usage may affect the recognition and action of RNA-editing enzymes at specific sites (Monian et al., 2022). As a result, the preference for certain codons can affect the frequency and location of RNA editing events. Therefore, the relationship between plant RNA editing and CUB is a promising area of investigation. Both processes have been implicated in gene expression and may contribute to plant adaptation. Understanding these interactions may provide crucial insights into the regulatory mechanisms underlying plant gene expression and evolution. Further research efforts encompassing diverse experimental approaches and sophisticated analyses are required to unravel the intricate relationship between RNA editing and CUB in the context of plant genetics.

Zingiberaceae plants exhibit rich species diversity and habitat variability from the equator to the mountains (Zhao et al., 2022). Certain species tolerate strong light, whereas others thrive in the shade, for instance, sister genera with distinct habitat preferences, such as *Roscoea* versus *Cautleya* and *Pommereschea* versus *Rhynchanthus*. The phylogenetic relationships of these four sister genera are (((*Roscoea*, *Cautleya*), (*Pommereschea*, *Rhynchanthus*)), outgroups) (**Supplementary Figure S1**) (Kress et al., 2002; Yang et al., 2021). *Roscoea* and *Pommereschea* grow primarily in open forests, meadows, and mountaintops and endure a certain degree of intense light. In contrast, *Cautleya* and *Rhynchanthus* grow epiphytically on shaded tree trunks or rocky streams, displaying shade adaptation. This study aimed to explore their adaptation to different habitats by investigating their codon preferences. In this study, we conducted a comprehensive comparative analysis of CUB in the cp genomes of four sister genera. Several CUB parameters, including the effective number of codons (ENc) and relative synonymous codon usage (RSCU), were estimated (Sharp et al., 2005; Angellotti et al., 2007). The primary objectives of this study encompass the following: 1) To investigate whether mutation pressure or selection pressure predominantly influences CUB in four taxonomic groups. 2) To determine if the optimal codon usage is correlated with habitat adaptation. 3) To explore the potential relationship between RNA editing and CUB.

## 2 Materials and methods

### 2.1 Sequence acquisition and filtering

The complete cp genome sequences of *Roscoea tibetica* (NC047420) were downloaded from GenBank (https://www.ncbi.nlm.nih.gov), and the complete cp genomes of *Cautleya gracilis*, *Pommereschea lackneri*, and *Rhynchanthus beesianus* (MW769781–MW769783) were assembled. We collected fresh leaves from three species in the wild (99.70°E, 24.18°N; 101.23°E, 21.99°N; 99.50°E, 22.48°N) and generated sequencing data for each species using the Illumina Hiseq 2500 platform. The sequencing data produced 45 gigabases of data for each species, with read counts of 277,483,161, 631,731,352, and 691,955,913, respectively. Subsequently, we performed *de novo* assembly of the raw data using the GetOrganelle tool with parameters (-R 15 -k 105,121; Jin et al., 2020). We annotated the coding sequences (CDS) using two different methods. Firstly, we selected complete and high-quality chloroplast genomes from closely related species within the Zingiberaceae family as references. The automatic annotation was performed using the GeSeq and DOGMA annotation tools (Wyman et al., 2004; Tillich et al., 2017). Secondly, we used chloroplast genomes of closely related species reported in the literature as reference sequences. The assembled chloroplast genomes were aligned with the reference sequences in Geneious software (Biomatters Ltd., Auckland, New Zealand) using the MAFFT tool (Katoh and Standley, 2013). Open reading frames were identified, and manual annotation for known genes was conducted based on similarity and positional information. To ensure accuracy, the consistency of coding sequences obtained from both annotation methods was further confirmed through BLAST searches. The accurately annotated CDS were then utilized for subsequent codon usage bias studies.

### 2.2 Content calculation and RSCU analysis

Perl programming scripts were used to calculate the GC content of the filtered CDSs, as well as the GC content at the first, second, and third positions of the codons (GC1, GC2, and GC3, respectively). Additionally, CodonW software (version 1.4.2, https://codonw.sourceforge.net/) was employed to calculate the RSCU, which represents the relative probability of specific codon usage among synonymous codons encoding the same amino acid (Sharp and Li, 1986). RSCU was determined by comparing the frequency of a codon with the average frequency of all codons encoding the corresponding amino acid. The RSCU value of a codon equals 1 if there is no codon preference. An RSCU value that exceeds 1 indicates a significant positive bias in codon usage. Conversely, an RSCU value less than 1 indicates a notable negative bias in codon usage (Sharp et al., 1993).

### 2.3 ENc and optimal codon analysis

The ENc refers to the number of codons effectively used in a gene (Wright, 1990). This reflects the deviation of codon usage from random selection and serves as an important indicator of the degree of synonymous CUB. The ENc values ranged from 20 (each amino acid used only one synonymous codon) to 61 (all synonymous codons were used equally). ENc and CAI values were calculated using the CodonW software (version 1.4.2) for the four species. Based on the ENc and CAI values, genes were rearranged, and the top and bottom 5% of genes were selected as high- and low-expression datasets, respectively. The optimal codons were determined using ΔRSCU with a threshold of 0.08. If the ΔRSCU value was > 0.08 and the absolute RSCU value was > 1, the codon was defined as optimal (Zhang et al., 2011).

### 2.4 ENc plot analysis

ENc-plot analysis was used to examine the codon usage patterns of genes. The expected ENc value was calculated using the following formula (Wright, 1990):


$$\text{ENc}=2+\text{GC}3_{\text{S}}+29/[\text{GC}3_{\text{S}}^{\;2}+{\left(1-\text{GC}3_{\text{S}}\right)}^{2}]$$

To explore the factors influencing CUB, an ENc-GC3s plot was generated, with GC3s and ENc on the x- and y-axes, respectively. GC3s are important nucleotide composition indicators that refer to the content of G and C at the third position of codons (excluding Trp, Met, and stop codons). If the CUB is primarily influenced by mutations, the genes will be located on or close to the standard curve. Conversely, if the CUB is influenced by natural selection, the genes will be located below the standard curve (Lü et al., 2005).

### 2.5 PR2-plot analysis

PR2-plot analysis is commonly used to estimate the effects of mutations and natural selection on CUB, specifically by analyzing the composition of the four bases at the third position of codons. Theoretically, the bases used at the third position of the codon should follow the PR2 rule (A=T and C=G). A 2D plot was constructed with A_3_/(A_3_+T_3_) and G_3_/(G_3_+C_3_) on the y- and x-axes, respectively (Sueoka, 1999). The vectors emanating from the center point (A=T, G=C) reflect the extent and direction of the base deviations (Wang et al., 2021). If the vectors are evenly distributed around the center point, mutations may entirely cause codon bias.

### 2.6 Neutrality plot analysis

Neutrality plot analysis was used to estimate the degree to which mutations and natural selection influenced CUB (Sueoka, 1988). A regression curve was plotted with GC12 (the average of GC1 and GC2) on the y-axis and GC3 on the x-axis. If GC12 is significantly correlated with GC3, and the slope of the regression curve is close to or equal to 1, the mutation is the primary factor affecting CUB. Conversely, if the slope is close to or equal to 0, then natural selection is the primary factor influencing CUB (Huo et al., 2021).

### 2.7 Determination of gene expression and RNA editing sites

Before the flowering period, in the afternoon under clear weather conditions, we collected leaves from plants in four locations (102.91°E, 24.76°N; 99.70°E, 24.18°N; 101.23°E, 21.99°N; and 99.50°E, 22.48°N) for these four species. We selected leaves from plants exhibiting normal growth and development. The leaves were rinsed with RNase-free water and dried with absorbent paper to remove any contaminants. Subsequently, the samples were quickly placed in liquid nitrogen for 30 minutes and then transferred for long-term storage at -80°C. For each plant population, we selected no fewer than three individuals with consistent growth as the source of biological replicate samples, aiming to minimize potential variations in results due to individual differences. The frozen samples were sent to Novogene Corporation for RNA extraction and transcriptome sequencing. These data are stored in NCBI under the accession number PRJNA997822, with the specific samples labeled as SRR25433125 to SRR25433136. The high-quality clean reads obtained were aligned to the reference sequences using the HISAT2 tool (Kim et al., 2019), and the resulting alignment results were visualized using the IGV tool (Robinson et al., 2023). The expression levels are typically quantified in Fragments Per Kilobase of transcript per Million fragments mapped (FPKM) values. The main process involved using the featureCounts function from the Rsubread library to process specified BAM files and GTF annotation files (Liao et al., 2014). It conducted read counting based on the mapping to genomic features, such as genes. The resulting read count information was stored in the fCountsList variable. Subsequently, the script utilized the rpkm function to calculate FPKM values from the obtained count data. To explore the potential relationship between RNA editing and CUB, the PREP-cp algorithm was employed to predict the RNA editing sites (Mower, 2009). A parameter threshold (cutoff value) of 0.8 was used to ensure prediction accuracy. The predicted editing sites were further validated by integrating transcriptomic data to confirm their authenticity.

## 3 Results

### 3.1 Nucleotide composition and RSCU analysis of the cp genes in four genera of sister plants

**Figure 1:**
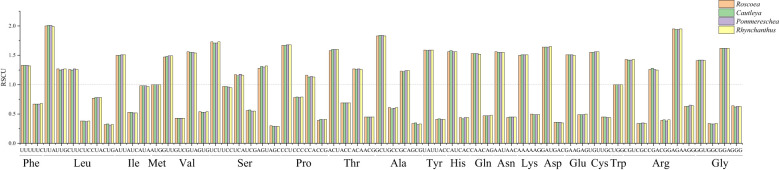
Relative synonymous codon use (RSCU) of chloroplast (cp) genes in four sister genera.

**Figure 2:**
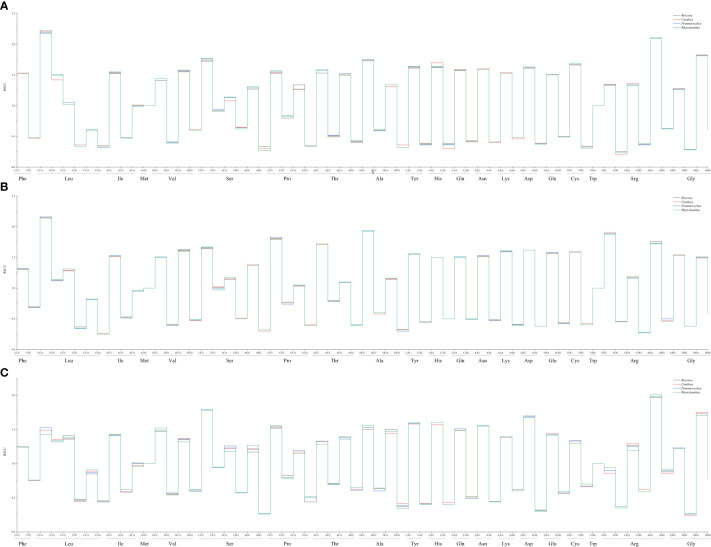
RSCU of photosynthesis-related genes in four sister genera **(A)**; RSCU of transcription- and translation-related genes in four sister genera **(B)**; RSCU of other genes in four sister genera **(C)**.

The overall nucleotide composition and codon usage at the third position of the codons were determined for the cp genes of the four sister plant species (*R. tibetica*, *C. gracilis*, *P. lackneri*, and *R. beesianus*). Among the four sister genera, the nucleotide T showed the highest usage, followed by A, G, and C. This uneven distribution of A, T, G, and C nucleotides indicated an A/T bias in the cp genomes. Based on their biological functions, the encoded genes were categorized as those related to photosynthesis, transcription, translation, or other miscellaneous functions. For photosynthesis-related genes, the nucleotide T had the highest usage, followed by A, C, and G. For transcription- and translation-related genes, the nucleotide A had the highest usage, followed by T, G, and C. Nucleotide usage of the other genes was consistent with the overall trend (**Supplementary Figure S2**). Additionally, compared with codons ending in G/C, cp genes showed a greater preference for codons ending in A/T, with a higher enrichment of A/T bases at the third codon position. The GC content (%) of the cp genes in the four sister species did not exceed 48%, and the GC content at different positions varied. Notably, both the overall GC content and GC content for the three types of genes followed the trend GC1 > GC2 > GC3, further indicating a bias toward A and T at the three codon positions (**Supplementary Figure S3**). RSCU (relative synonymous codon usage) analysis of the codons revealed that all four sister genera had 29 synonymous codons with relative usage rates > 1 (**Figure 1**), of which seven codons were overrepresented (RSCU > 1.6). Both photosynthesis-related and transcription- and translation-related genes had nine overrepresented codons, while four codons were overrepresented in the other gene category (**Figure 2**). The average ENc (effective number of codons) values for both the four sister genera and photosynthesis-related genes ranged between 46 and 47. In contrast, the average ENc values for transcription- and translation-related genes ranged between 45 and 46, whereas for other genes, the average ENc values were between 49 and 50. These results indicated the presence of certain codon preferences.

### 3.2 ENc-plot analysis

**Figure 3:**
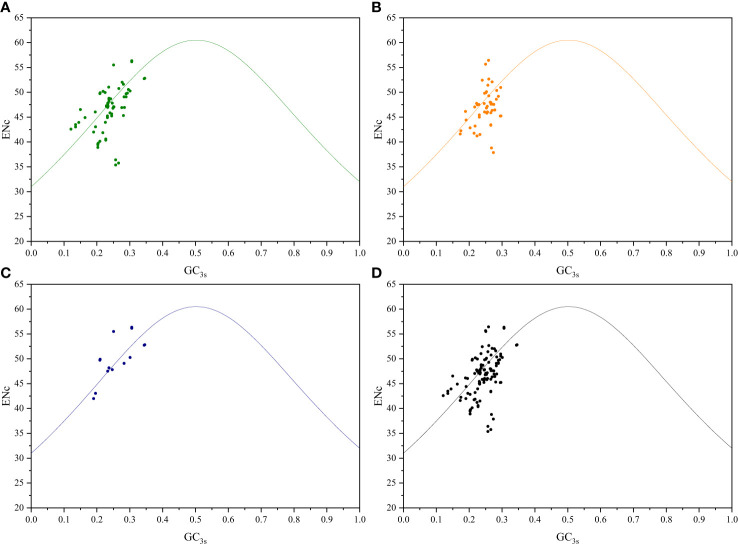
ENc-plot analysis of *Roscoea* and *Cautleya* cp genes. **(A)** represents genes related to photosynthesis, **(B)** represents genes related to transcription and translation, **(C)** represents other genes, and **(D)** represents all genes.

To further investigate the relationship between nucleotide composition and codon usage preference, ENc-plot analysis was conducted to study the factors influencing codon usage, including mutation pressure and natural selection. If mutation pressure is the primary factor influencing codon usage preference, the real ENc values should align closely with the ENcexp curve. Conversely, if natural selection is the primary factor affecting the codon usage preference, the real ENc values will deviate from the ENcexp curve, thus positioning below the curve (Gao et al., 2022). Among both sister genera groups, certain genes related to photosynthesis, transcription, and translation were distributed below the standard curve, with ENc values between 35 and 57, thus indicating a certain level of CUB (**Figure 3**; **Supplementary Figure S4**). In addition to mutations, natural selection may influence the variability in codon usage.

### 3.3 PR2-plot analysis

**Figure 4:**
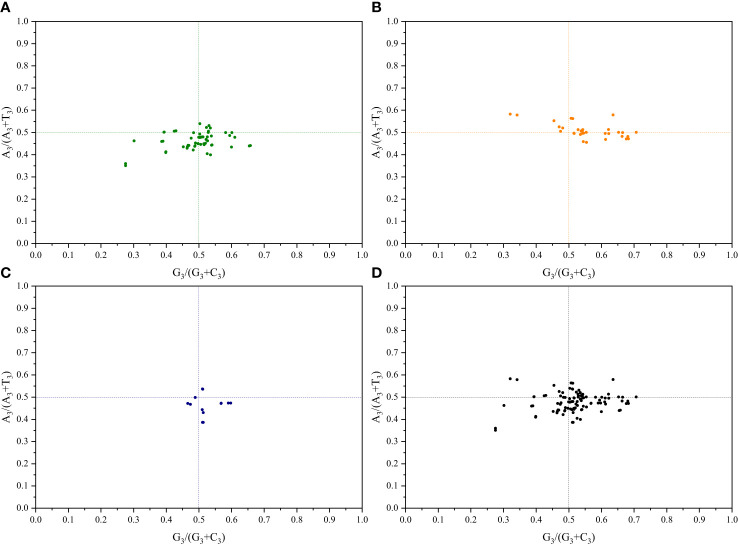
PR2-plot analysis of *Roscoea* and *Cautleya* cp genes. **(A)** represents genes related to photosynthesis, **(B)** represents genes related to transcription and translation, **(C)** represents other genes, and **(D)** represents all genes.

To understand the impact of mutations and selection pressures on codon usage in cp genes, a parity rule 2 (PR2) bias plot analysis was conducted to explore the relationship between the third base of codons. Equal nucleotide usage frequencies indicate that only mutation pressure influences CUB (Kawabe and Miyashita, 2003). Conversely, natural selection may enable the unequal usage of A and T with G and C bases (Sueoka, 1995). Herein, for both pairs of sister genera, the A_3_/(A_3_+T_3_) ratio of genes related to photosynthesis primarily showed a distribution < 0.5, suggesting a higher frequency of T bases than that of A bases. Additionally, the G_3_/(G_3_+C_3_) ratio showed a distribution of 0.5, indicating a higher frequency of G bases than that of C bases. Regarding genes related to transcription and translation, the A_3_/(A_3_+T_3_) ratio for both pairs of sister genera primarily showed a distribution of 0.5, indicating a higher frequency of A bases than that of T bases. The G_3_/(G_3_+C_3_) ratio, in contrast, predominantly showed a distribution > 0.5, indicating a higher frequency of G bases than that of C bases (**Figure 4**; **Supplementary Figure S5**). These results further demonstrate that both base mutations and natural selection jointly influence codon usage preferences in the cp genomes of the two pairs of sister genera, with natural selection primarily affecting codon usage preference.

### 3.4 Neutrality plot

**Figure 5:**
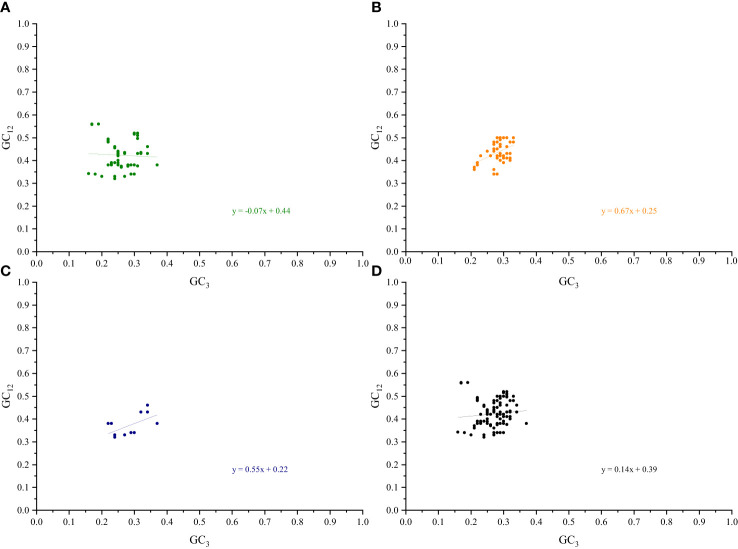
Neutrality plot analysis of *Roscoea* and *Cautleya* cp genes. **(A)** represents genes related to photosynthesis, **(B)** represents genes related to transcription and translation, **(C)** represents other genes, and **(D)** represents all genes.

A separate neutral plot analysis of the cp genes of the two pairs of sister genera was conducted to investigate the relationships between the bases at the three codon positions. No significant correlations between the GC12 and GC3 genes related to photosynthesis were found in *Roscoea* and *Cautleya*, indicating that natural selection may influence their CUB (Nie et al., 2012). A similar phenomenon was observed for *Pommereschea* and *Rhynchanthus*. Neutral plot analysis further explained the roles of mutation pressure and natural selection. When the slope of the regression curve is approximately 0, natural selection plays a dominant role in the CUB, whereas a slope of approximately 1 indicates the dominance of mutation pressure (Butt et al., 2016; Zhao et al., 2016). For genes related to photosynthesis, transcription and translation, other genes, and all genes in *Roscoea* and *Cautleya*, the absolute values of the slopes of the regression curves were 0.07, 0.67, 0.55, and 0.14, respectively. Similar results were obtained for *Pommereschea* and *Rhynchanthus* with absolute values of the regression curve slopes of 0.09, 0.62, 0.50, and 0.10, respectively (**Figure 5**; **Supplementary Figure S6**). These results further indicate that natural selection primarily influences codon usage patterns in the cp genomes of the two pairs of sister genera, particularly in genes related to photosynthesis, whereas base mutations play a secondary role.

### 3.5 Optimal codon determination

To determine the optimal codons in the protein-coding sequences of the cp genomes of the four sister genera, genes were sorted based on their ENc and CAI values. The top and bottom 5% of the sequences were selected within their corresponding ENc and CAI value ranges as high- and low-expression gene samples, respectively. Using previously calculated RSCU values for all synonymous codons, 10, 11, 11, and 9 optimal codons were identified for *Roscoea*, *Cautleya*, *Pommereschea*, and *Rhynchanthus*, respectively. The majority of optimal codons ended with A/U (except for UUG). Six optimal codons were shared among genera: CUU (Leu), GUU (Val), GUA (Val), UCA (Ser), ACA (Thr), and GCA (Ala). These findings suggest a preference for A/U at the third position of their codons. Notably, the two optimal codons (AGU and GAU) were present only in *Roscoea* and *Pommereschea* (**Table 1**), which inhabit similar environments. Therefore, the presence of these codons may be related to their adaptation to similar habitats.

**Table 1:** Optimal codons of chloroplast genes in four sisters genera.

		*Roscoea*		*Cautleya*		*Pommereschea*		*Rhynchanthus*	
		RSCU	ΔRSCU	RSCU	ΔRSCU	RSCU	ΔRSCU	RSCU	ΔRSCU
Leu	UUG			1.25	0.25				
	CUU	1.26	0.51	1.25	0.38	1.27	0.62	1.26	0.70
Val	GUU	1.47	0.43	1.48	0.40	1.49	0.37	1.49	0.53
	GUA	1.56	0.36	1.55	0.35	1.55	0.43	1.54	0.16
Ser	UCA	1.17	0.77	1.15	1.03	1.18	0.59	1.16	0.67
	AGU	1.28	0.22			1.30	0.23		
Pro	CCU	1.67	0.32			1.68	0.30	1.68	0.09
	ACA					1.27	0.58		
Thr	ACA	1.27	0.83	1.26	0.56			1.26	0.60
Ala	GCA	1.23	0.66	1.22	0.76	1.24	0.68	1.24	0.63
Tyr	UAU			1.58	0.11	1.59	0.11	1.59	0.14
Asp	GAU	1.64	0.17			1.64	0.09		
Cys	UGU	1.55	0.24						
Arg	AGA			1.94	0.38				
Gly	GGU			1.42	0.26	1.42	0.26	1.41	0.32
	GGA			1.62	0.23				

### 3.6 Relationship between codon usage and gene expression

**Figure 6:**
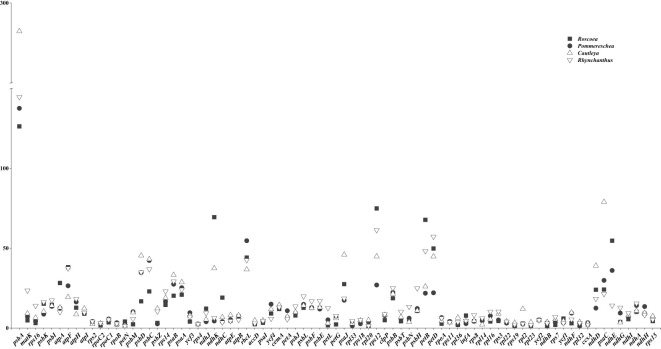
Expression of cp genes in four sister genera.

To gain a better understanding of the relationship between codon usage and gene expression, the expression levels of cp genes were obtained from the section 2.7, as detailed in the Materials and Methods section. Through a comparison of gene expression levels across the four taxa, we identified eight coding genes with differential expression in different environments. Among the seven genes analyzed, *Cautleya* and *Rhynchanthus* exhibited higher expression levels of *psbA*, *psaB*, *ndhI*, *matK*, *rps3*, *rps7*, and *rps14* than those of *Roscoea* and *Pommereschea*, whereas *Roscoea* and *Pommereschea* showed higher expression levels of *rbcL* than those for *Cautleya* and *Rhynchanthus* (**Figure 6**). The ENc values for these eight genes were < 50, indicating a relatively strong CUB. In addition, the third base of these codons typically ended in A/U (**Supplementary Figure S7** and **Supplementary Table S1**). Notably, the *psbA* gene contained no codon for Leu.

Furthermore, the RNA editing of these eight genes in the four sister genera were investigated. PREP-cp prediction was used and validated using transcriptome data (Mower, 2009), resulting in the identification of 20 authentic RNA editing sites among the five genes, all involving C-to-U transitions. Among them, 20% (4 sites), 65% (13 sites), and 15% (3 sites) occurred at the first, second, and third codon positions, respectively. Moreover, 40% (8 sites) of the RNA editing events resulted in the conversion of neutral amino acids to hydrophobic amino acids, whereas another 40% (8 sites) led to the conversion of hydrophobic amino acids to other hydrophobic amino acids. Additionally, 10% (2 sites) of the RNA editing events caused basic amino acids to change into neutral amino acids, and the remaining 10% (2 sites) involved basic amino acids being converted into other basic amino acids or hydrophobic amino acids turning into neutral amino acids (**Supplementary Table S2**). We predicted RNA editing sites for these eight genes and observed a preference for RNA editing to occur in codons with high RSCU values in these genes.

## 4 Discussion

### 4.1 Base composition

In the study of CUB in plant cp genomes, base composition analysis is an essential aspect that involves investigating the frequency of AGCT nucleotides, the GC content, and their relationship with codon usage preference. Valuable information regarding gene expression and evolution can be obtained by analyzing the codons and bases in cp genomes (Southworth et al., 2018; Chakraborty et al., 2019; Shen et al., 2020). Moreover, certain studies have suggested that biases in the base composition of the genome may cause mutation pressure and influence codon usage preferences via an appropriate balance of G + C nucleotide pairs (Li et al., 2002). In this study, the cp genes of two pairs of sister genera that exhibited significant habitat differentiation were analyzed, and their codon usage preferences and patterns influencing CUB evolution were explored.

Herein, the overall frequency of codon usage for A and T was significantly higher than that for G and C. All gene-coding sequences were enriched with AT bases. In the cp genes of the four sister genera, codons ending with A and T were preferred, with T being the most common nucleotide in genes associated with photosynthesis. Among the cp genes of the sister genera, the GC content was highest in the first position of the codon and lowest in the third. Previous studies reported that codons preferentially ended with A or T in angiosperm nuclear genomes, whereas they typically ended with G or C in monocot genes (Kawabe and Miyashita, 2003). A highly conserved GC content in the coding sequences of cp genes in these two pairs of sister genera were observed, suggesting that evolutionary equilibrium was attained over time. The usage frequency of pyrimidines (C and T) was higher than that of purines (A and G) in the third position of their codons. Similar results have been reported in previous studies (Zhang et al., 2013). Additionally, certain studies have proposed that codon usage preferences are primarily influenced by differences in G and C content, rather than differences in A and T (Zhang et al., 2007).

Generally, the term “optimal codon” refers to the codons that efficiently facilitate translation and protein synthesis under specific environmental conditions. Research findings suggest that CUB can influence the speed and accuracy of gene expression in cps, thereby affecting cellular energy utilization and genome complexity (Lane and Martin, 2010). Furthermore, codon selection may have played a role in the evolution of cp genomes. A preference for A/U as the ending nucleotide in optimal codons was observed, and shared optimal codons in similar habitats were identified, thus indicating a potential association with their adaptation to the environment. In addition, optimal codons can be used to design degenerate primers and introduce site mutations in evolutionary studies.

### 4.2 Factors influencing CUB

The formation of codon bias is a result of mutations, and the bias in mutations is shaped by the underlying mutational mechanisms. Mutational selection is neutral (it does not affect normal mechanisms) and acts equally on all DNA sequences of the organism. In most organisms, the frequency of TA dinucleotides is lower than expected based on random nucleotide usage. This phenomenon is a consequence of avoiding the use of termination codons, such as TAA and TAG, just as mRNA avoids using UA because it is susceptible to ribonuclease activity (Bulmer, 1991). Numerous base mutations are caused by random mutations, mispairing during repair, replication errors, or methylation.

Unlike pure mutational mechanisms, selective pressure influences the use of synonymous codons. Codon bias that results from selective pressure may exist in specific genes or positions within codons to enhance translation efficiency, accuracy, or protein-folding dynamics. At the RNA level, selective pressure for efficient transcription has been reported, where rich nucleotides improve mRNA translation efficiency (Xia, 2005). Selection also occurs at the mRNA level and affects mRNA folding and decay through preferential selection. This suggests the presence of overall optimization to reduce the time of action of the ribosome during mRNA translation, thereby enhancing its efficiency. The use of optimal codons can influence translation speed and elongation rate, thus affecting protein-folding co-translational processes (Kawabe and Miyashita, 2003). In large populations of effective codons, organism selection plays a significant role in translational optimization (Bulmer, 1987).

Herein, both natural selection and mutations influenced the CUB of cp genes in the two pairs of sister genera, with natural selection primarily shaping codon usage patterns, particularly for genes related to photosynthesis, while base mutations played a secondary role. This is consistent with previous research on *Panicum*, *Malus*, and Euphorbiaceae (Wang et al., 2020; Li et al., 2021; Li et al., 2023). Notably, the factors influencing CUB are highly complex, and other factors, such as gene length, gene expression levels, recombination rates, and genetic code repair, may also play a role (Marais et al., 2001; Barbhuiya et al., 2021; Huang et al., 2022).

### 4.3 CUB and gene expression and RNA editing

Codon usage patterns in cp genomes are associated with environmental adaptation. Chloroplast genomes are the plant organelles responsible for photosynthesis and energy conversion, therefore, the proteins encoded by the cp genome are closely related to these functions. Research indicates that CUB in the cp genome is also linked to the adaptability of cps to their environment and efficiency of gene expression (Zhang et al., 2007).

Significant differences in the expression levels of eight genes were found between the two sister genera in similar habitats, and the ENc values showed strong preferences. Among them, the *psbA*, *psaB*, *rbcL*, and *ndhI* genes are directly involved in the regulation of photosynthesis, including light capture, photoprotection, stress adaptation, and redox reactions (Jensen et al., 2000; Adams et al., 2001; Munekage et al., 2002; Heinemeyer et al., 2004; Nishizawa et al., 2006; Takahashi et al., 2007; Whitney and Sharwood, 2008; Sage and Sage, 2009). In certain studies, the *rps14*, *rps3*, and *rps7* genes have been suggested to participate in translation. In addition, their expression levels may be regulated by light, temperature, and other environmental factors, implying their potential roles in photosynthesis and plant growth and development regulation (Barkan, 2011; Shanmugabalaji et al., 2013; Dent et al., 2015; Wang et al., 2017). However, the role of the *matK* gene in plant light adaptation has not been extensively studied. To date, most studies have focused on the phylogeny and species identification of *matK* gene. Certain studies have used cp genome data from multiple plant species, including the *matK* gene, to analyze the phylogenetic relationships and evolutionary patterns of plant species, which may be related to their photosynthetic adaptation ability (Jansen et al., 2007; Timme et al., 2007). Therefore, the differential expression of these genes may be associated with their adaptation to different habitats, particularly in response to light conditions.

RNA editing in the cp genome of plants is a post-transcriptional modification process that involves changing the bases in the transcribed RNA sequence to other bases, thereby introducing diversity between the transcript and genomic sequences (Ichinose and Sugita, 2016). This type of RNA editing is widespread in plants and plays crucial roles in regulating gene expression, increasing functional diversity, and adapting to environmental changes. Studies have suggested that plants utilize RNA editing to adapt to different environmental pressures, such as temperature, light, and salinity. Adaptive RNA editing can confer survival advantages to plants in different habitats (Wang et al., 2015). In addition, RNA editing plays a significant role in regulating the expression of genes involved in photosynthesis. The present study found that in differentially expressed genes, RNA editing resulted in more hydrophobic amino acid changes, thus significantly increasing the hydrophobicity of the edited proteins. This pattern is similar to the changes observed in RNA editing sites in cotton cp genes, which may favor the formation of stable protein structures. When hydrophilic mutations occur in the core of a protein structure, they can cause severe instability or even loss of folding and function (De Vos et al., 2001). RNA editing appears to be more likely to occur in codons with high RSCU values. However, further data analysis and experimental validation are required to investigate whether there is a mutual influence between RNA editing and CUB and to understand the mechanisms underlying this potential interaction.

## 5 Conclusions

This study investigated the codon usage patterns of the cp genomes in two pairs of sister genera of the Zingiberaceae family that showed clear habitat differentiation. The relationship between CUB and gene expression was also explored. High CUBs were observed, with protein-coding genes and the third position of codons enriched in A/T bases. Different patterns of codon usage were observed among the different gene types. Natural selection likely plays a considerable role in shaping the CUB in the cp genes of species from different habitats, whereas mutations play a secondary role. Nine to 11 optimal codons were identified in the cp genome, with two showing similarities between species from the same habitat. Genes related to light adaptation exhibited differential expression in species from different habitats, and RNA editing is likely to occur in codons with higher RSCU values. Future research into the potential associations between CUB and RNA editing, as well as multidimensional analyses of factors influencing CUB, is necessary.

## Data availability statement

The datasets presented in this study can be found in online repositories. The names of the repository/repositories and accession number(s) can be found below: https://www.ncbi.nlm.nih.gov/genbank/, PRJNA997822.

## Author contributions

QY: Conceptualization, Software, Writing – original draft. CX: Software, Writing – review & editing. Q-SX: Resources, Writing – review & editing. Y-TL: Data curation, Writing – review & editing. LL: Resources, Writing – review & editing. J-LZ: Conceptualization, Funding acquisition, Writing – review & editing.

## Supplementary material

Supplementary Figure 1 The evolutionary relationship of four sisters genera (*Roscoea*, *Cautleya*, *Pommereschea*, and *Rhynchanthus*).

Supplementary Figure 2 Comparison of the base composition of four sister genera with their cp genes. **(A)** is all genes, **(B)** is photosynthesis-related genes, **(C)** is transcription- and translation-related genes, and **(D)** is other genes. The y-coordinate represents the frequency of base usage.

Supplementary Figure 3 Content of the cp genes GC, GC1, GC2, and GC3 in four sister genera. **(A)** is all genes, **(B)** is photosynthesis-related genes, **(C)** is transcription- and translation-related genes, and **(D)** is other genes. The y-coordinate represents the GC content.

Supplementary Figure 4 ENc-plot analysis of Pommereschea and Rhynchanthus cp genes. A represents genes related to photosynthesis, B represents genes related to transcription and translation, C represents other genes, and D represents all genes.

Supplementary Figure 5 PR2-plot analysis of Pommereschea and Rhynchanthus cp genes. A represents genes related to photosynthesis, B represents genes related to transcription and translation, C represents other genes, and D represents all genes.

Supplementary Figure 6 Neutrality plot analysis of Pommereschea and Rhynchanthus cp genes. A represents genes related to photosynthesis, B represents genes related to transcription and translation, C represents other genes, and D represents all genes.

Supplementary Figure 7 Relative synonymous codon use (RSCU) of differentially expressed genes. **(A)** is *psbA*, **(B)** is *psaB*, **(C)** is *rbcL*, **(D)** is *ndhI*, **(E)** is *matK*, **(F)** is *rps14*, **(G)** is *rps3*, and **(H)** is *rps7*.
