# Supplementary figures and images for: Codon usage bias in chloroplast genes implicate adaptive evolution of four ginger species
Source: Front Plant Sci. 2023 Dec 15;14:1304264. doi: 10.3389/fpls.2023.1304264 (PMC10758403; doi:10.3389/fpls.2023.1304264)

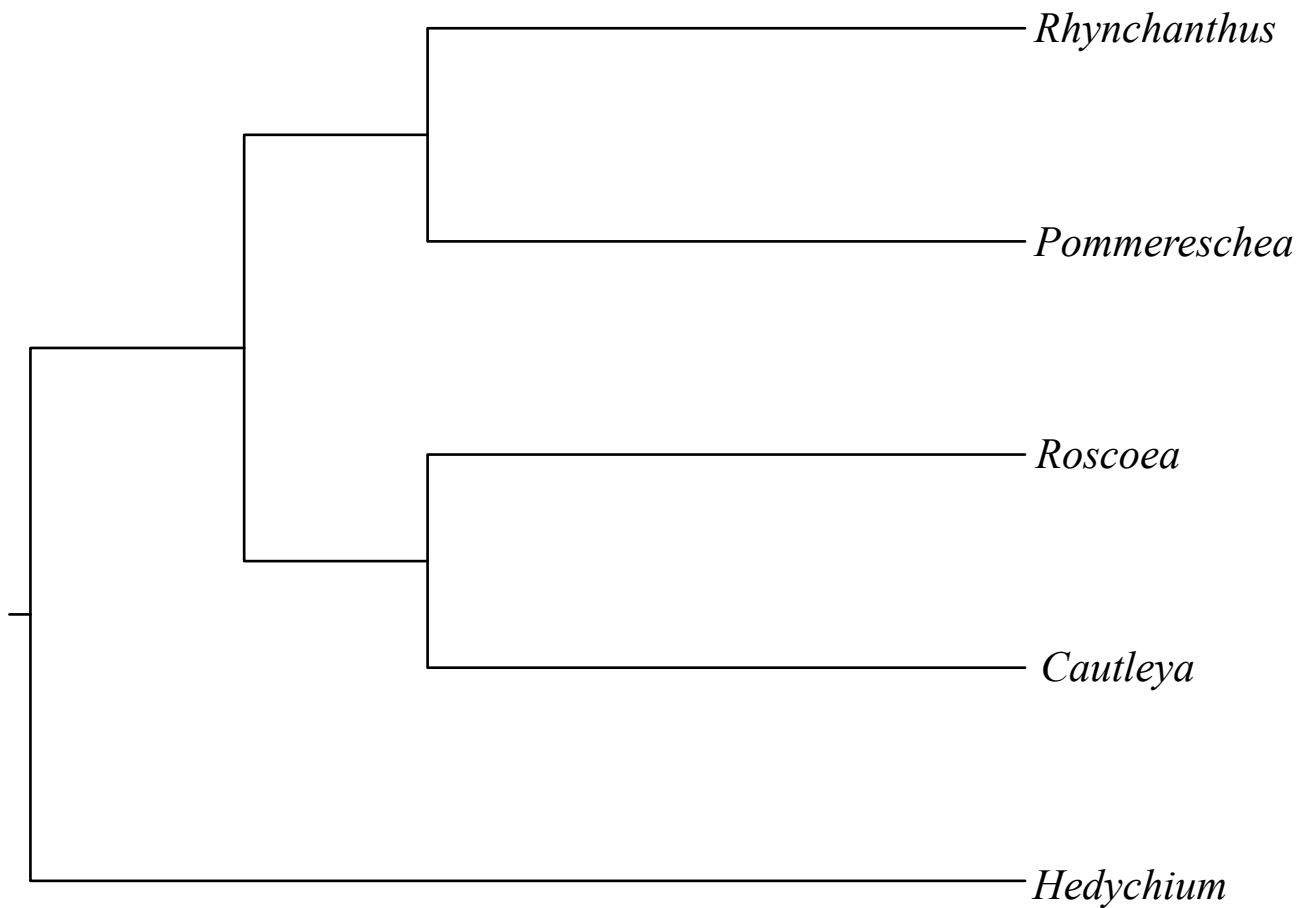

Supplement: Supplementary Figure 1 — The evolutionary relationship of four sisters genera (Roscoea, Cautleya, Pommereschea, and Rhynchanthus). [file DataSheet_1.zip › Supplementary Files/Figure S1.pdf]

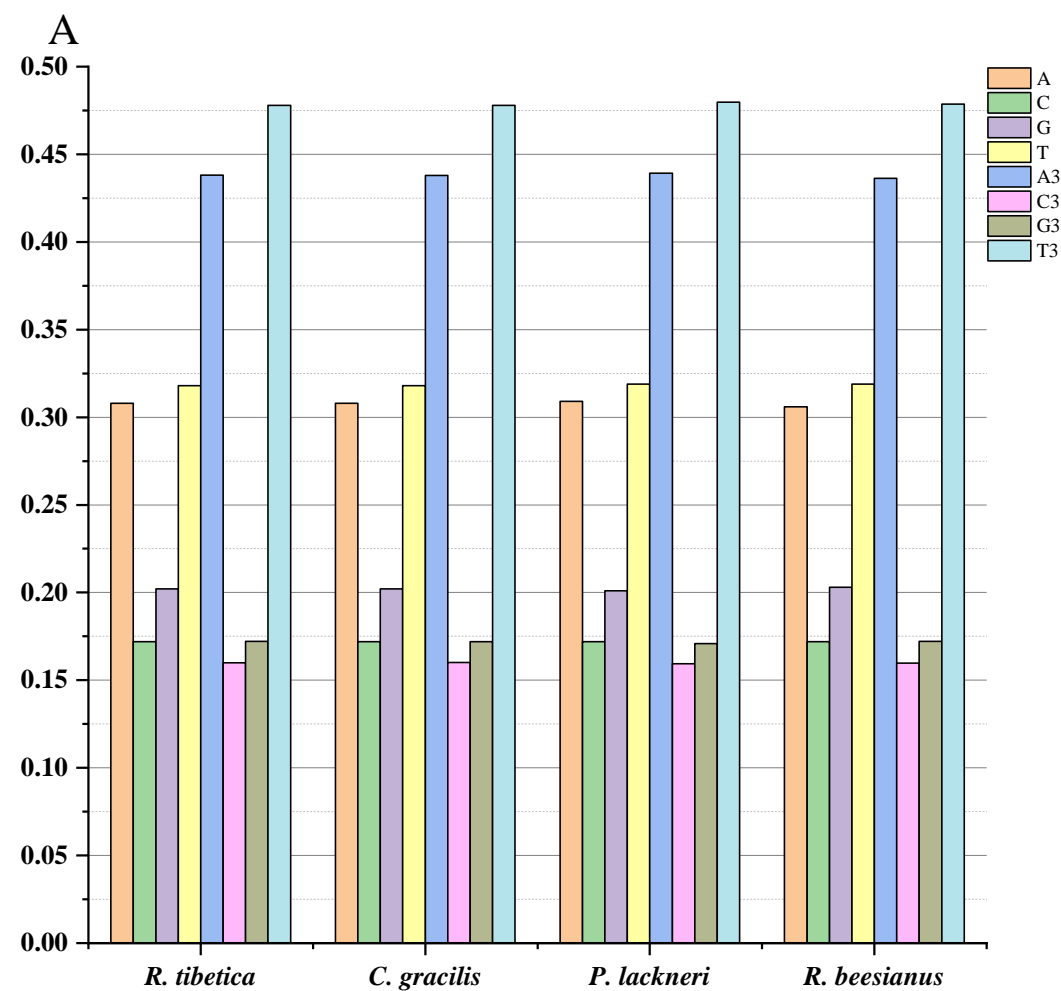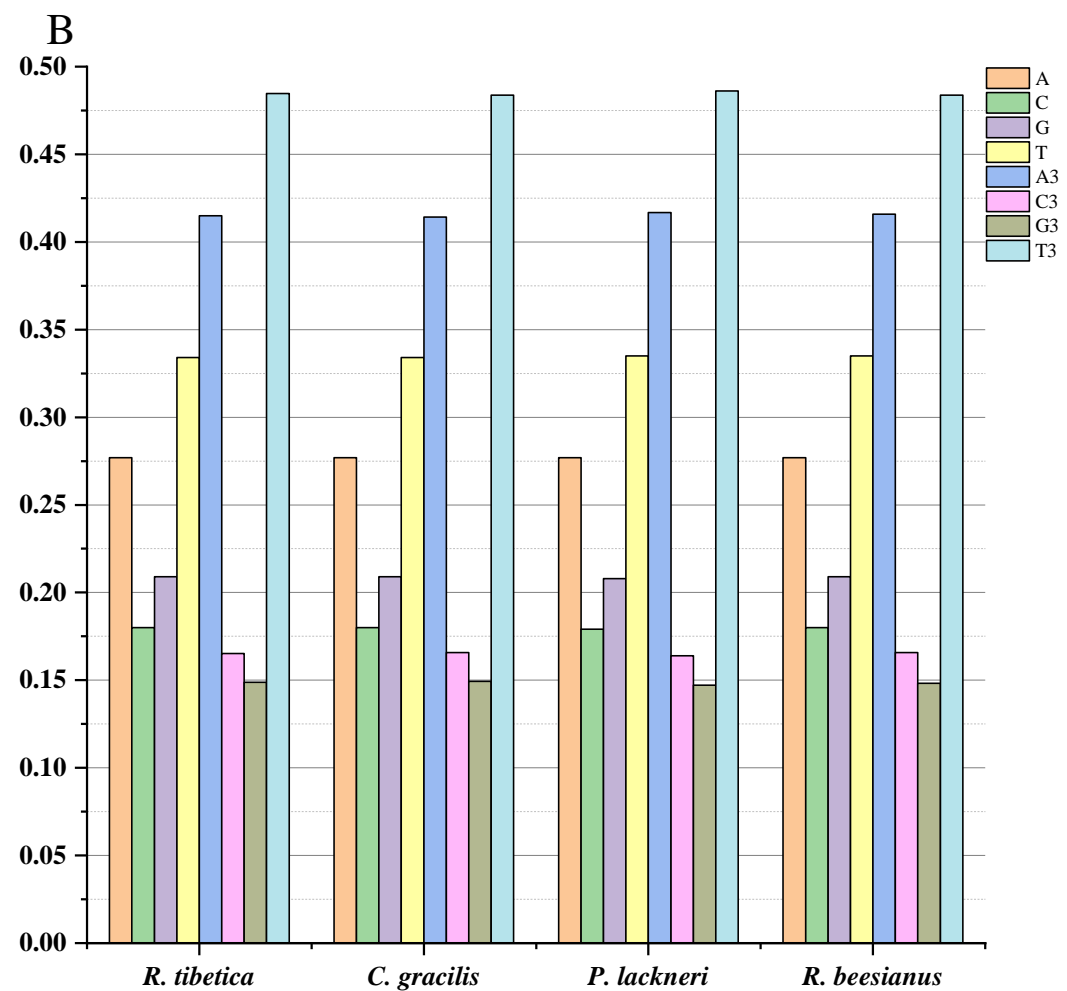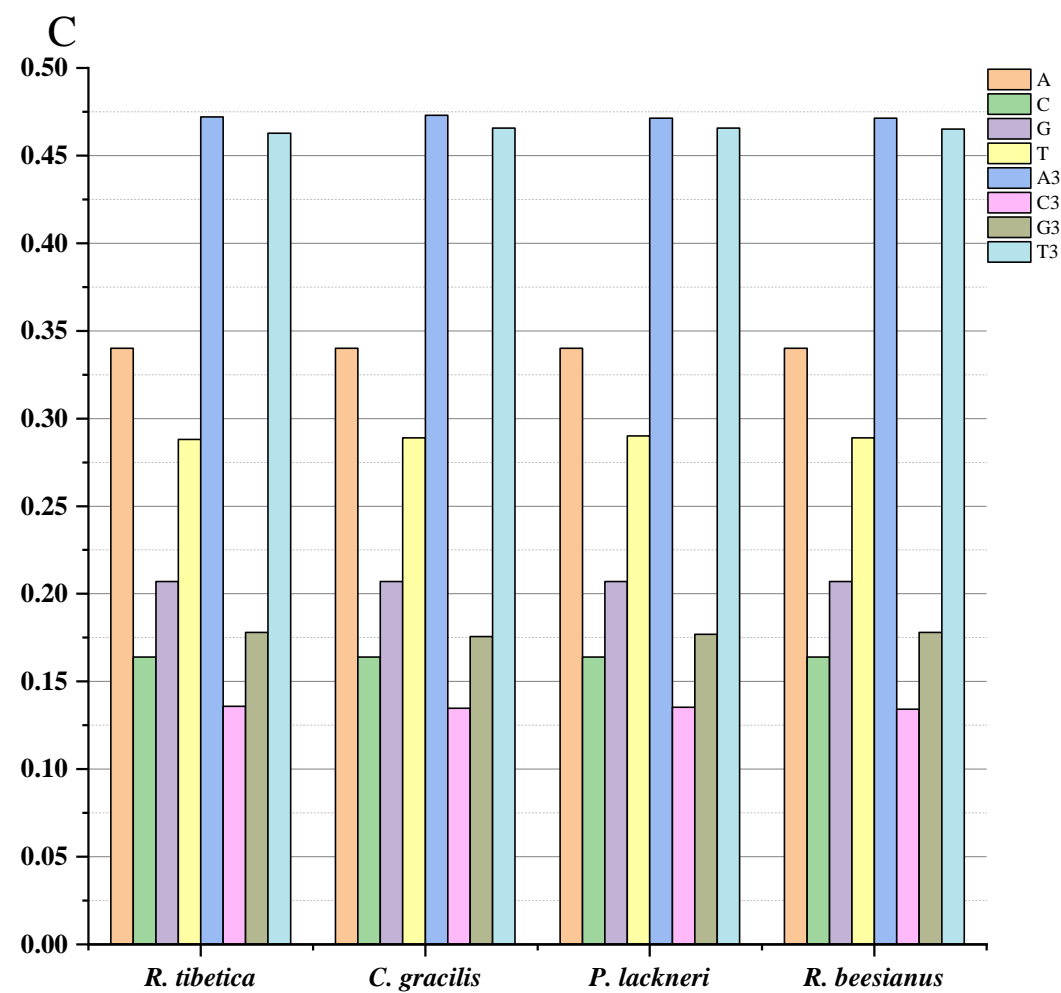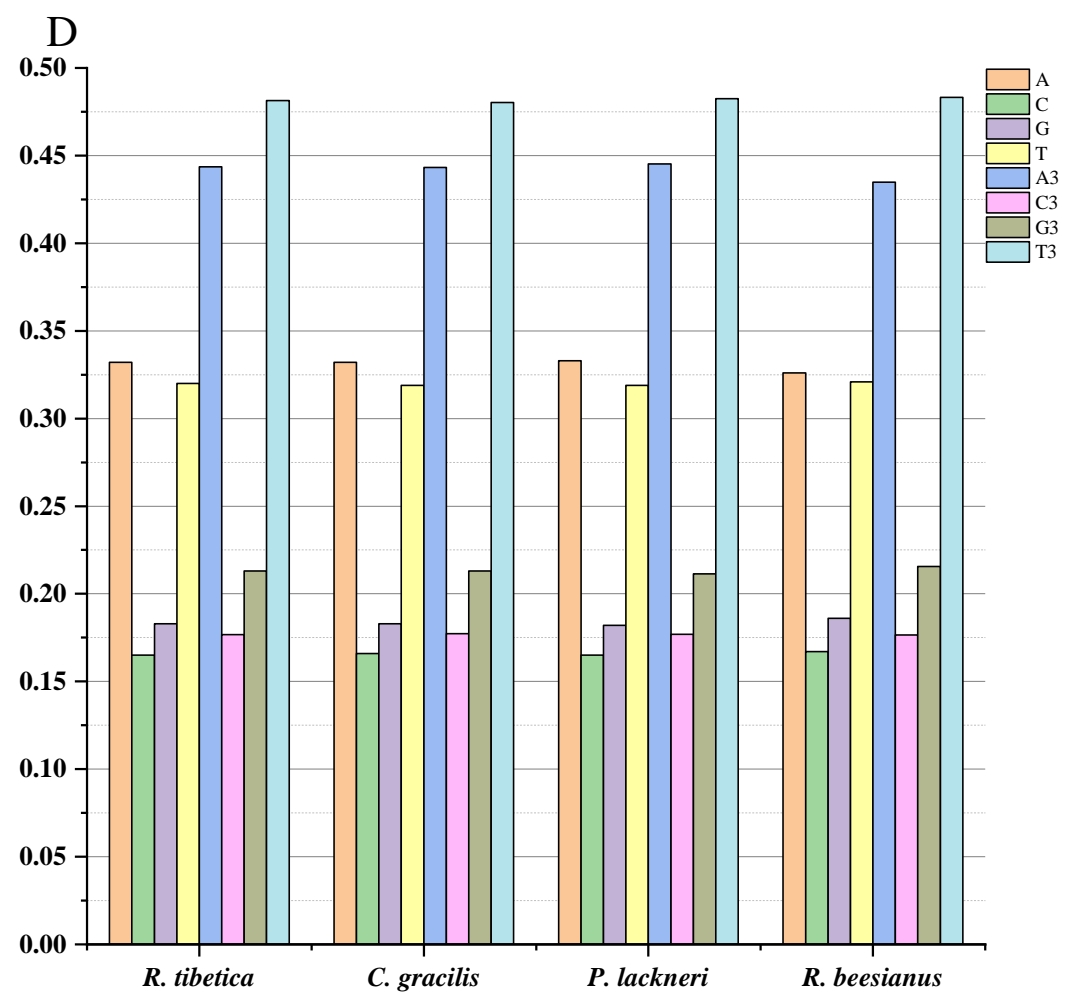

Supplement: Supplementary Figure 1 — The evolutionary relationship of four sisters genera (Roscoea, Cautleya, Pommereschea, and Rhynchanthus). [file DataSheet_1.zip › Supplementary Files/Figure S2.pdf]

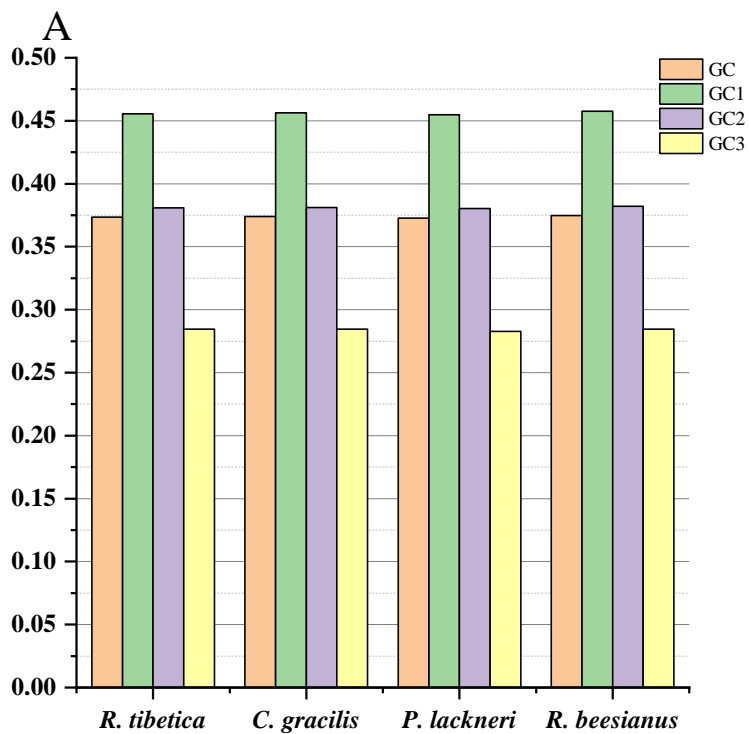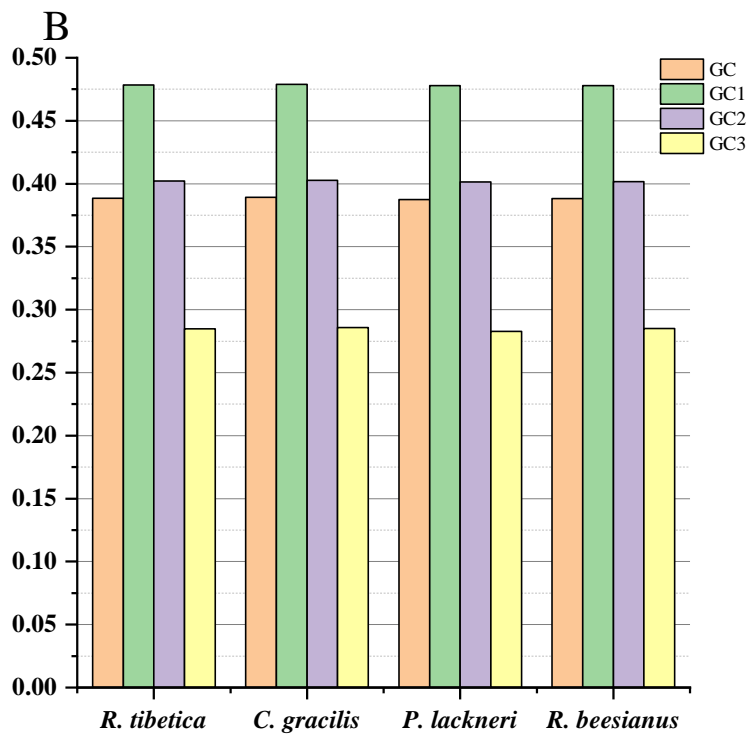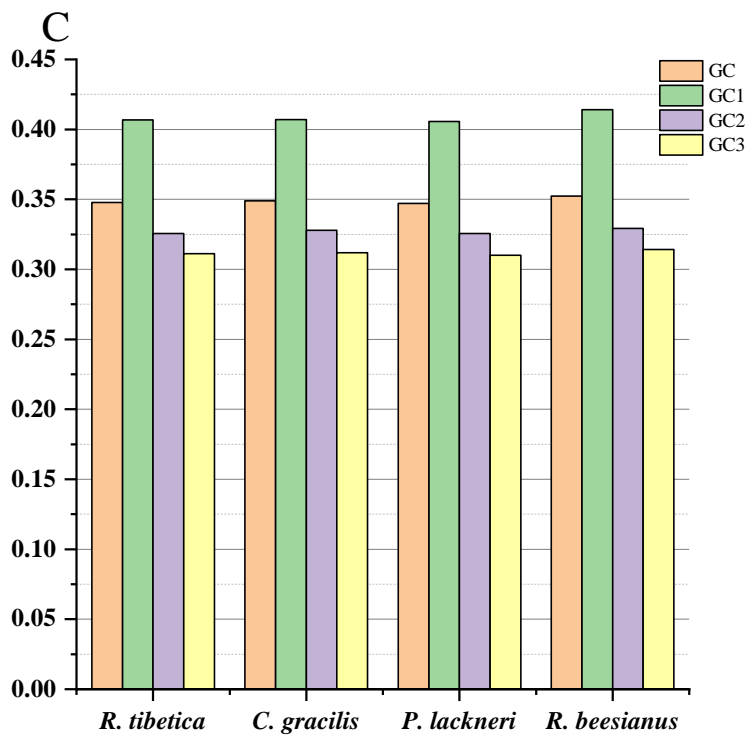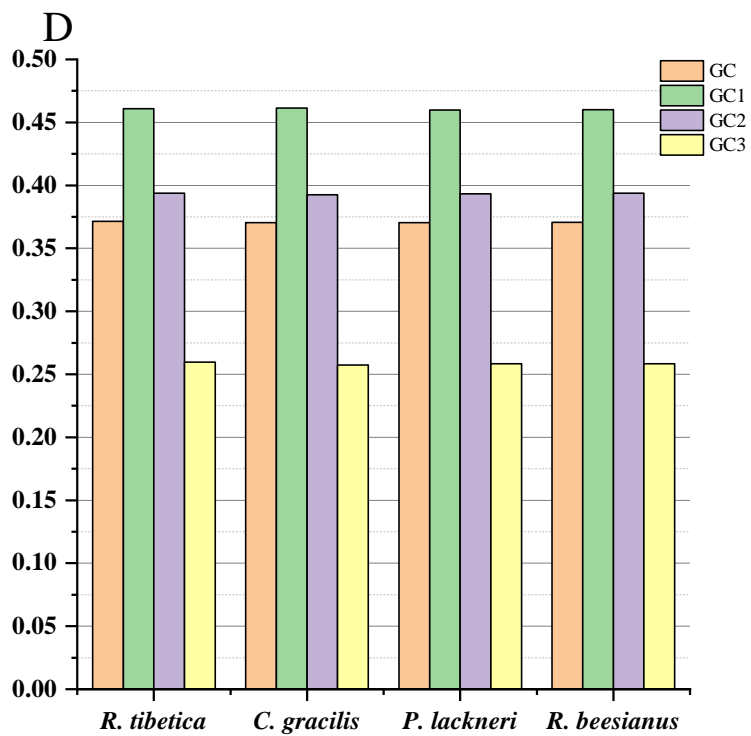

Supplement: Supplementary Figure 1 — The evolutionary relationship of four sisters genera (Roscoea, Cautleya, Pommereschea, and Rhynchanthus). [file DataSheet_1.zip › Supplementary Files/Figure S3.pdf]

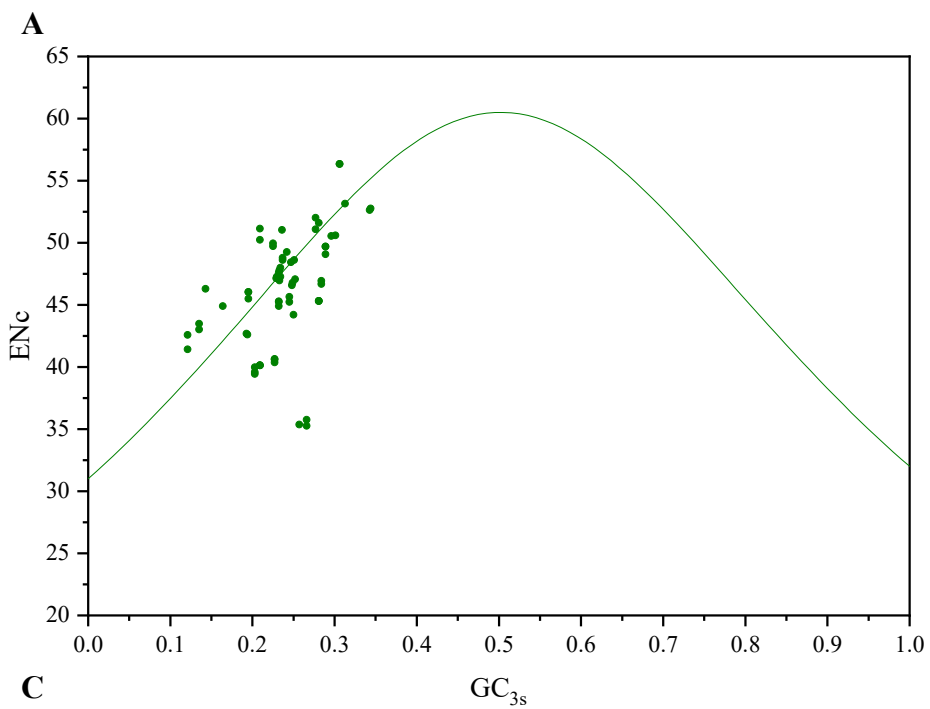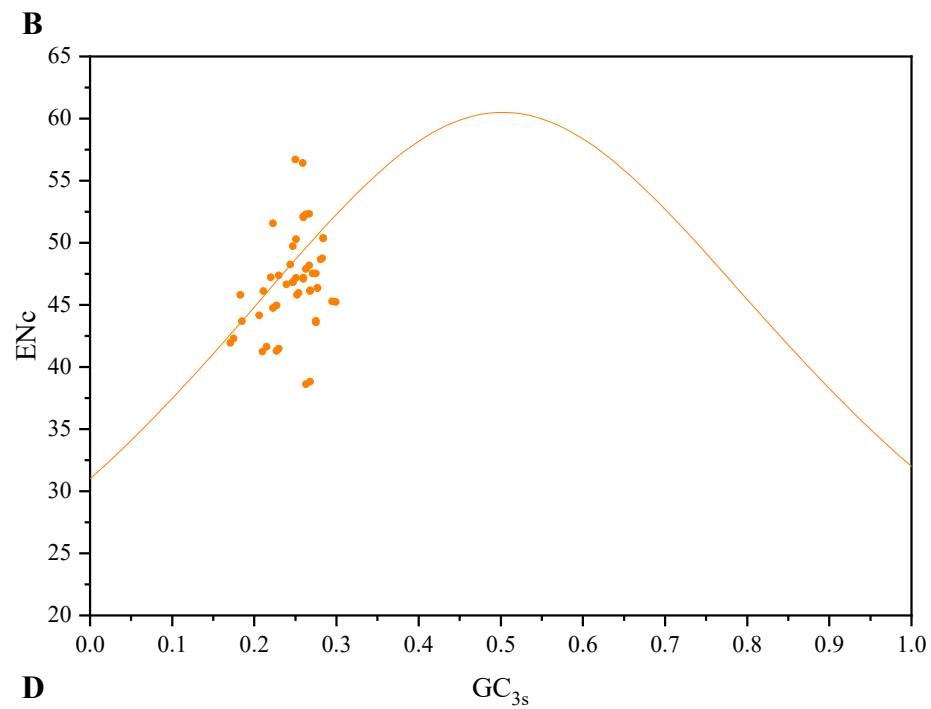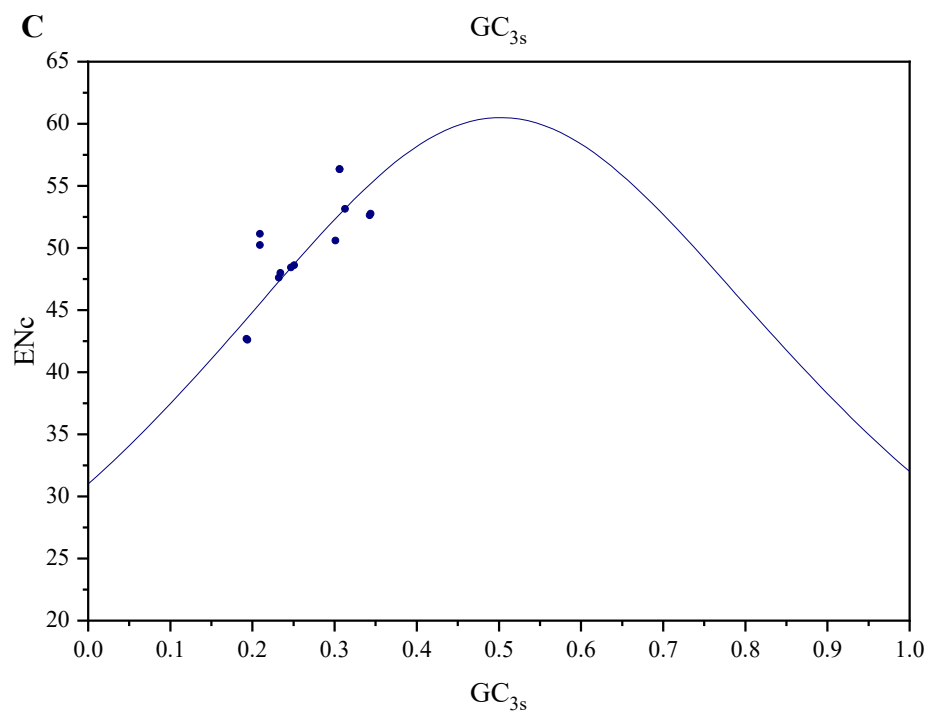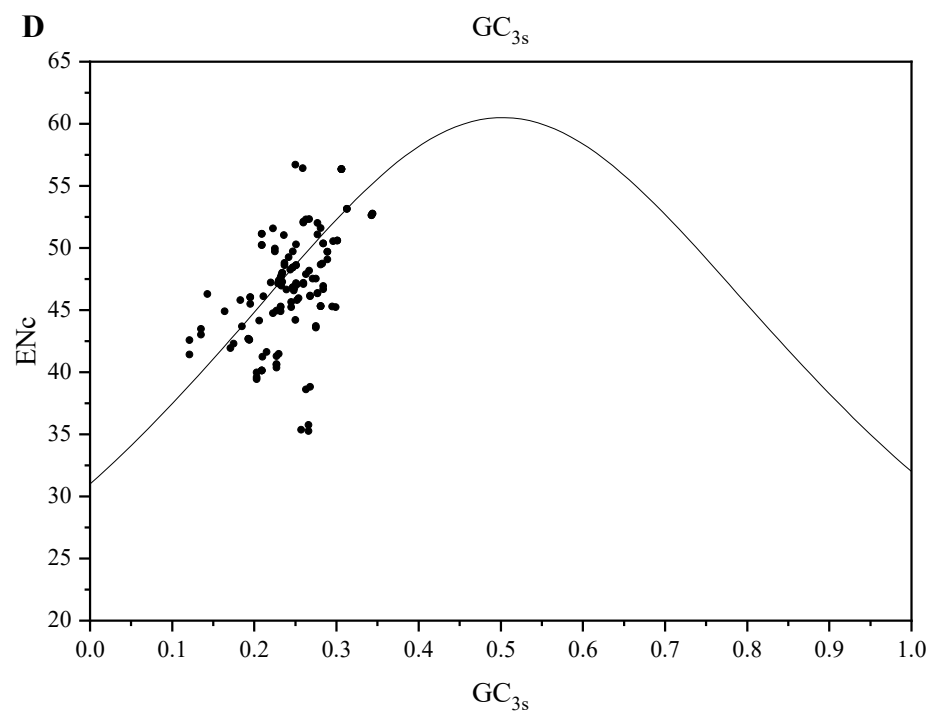

Supplement: Supplementary Figure 1 — The evolutionary relationship of four sisters genera (Roscoea, Cautleya, Pommereschea, and Rhynchanthus). [file DataSheet_1.zip › Supplementary Files/Figure S4.pdf]

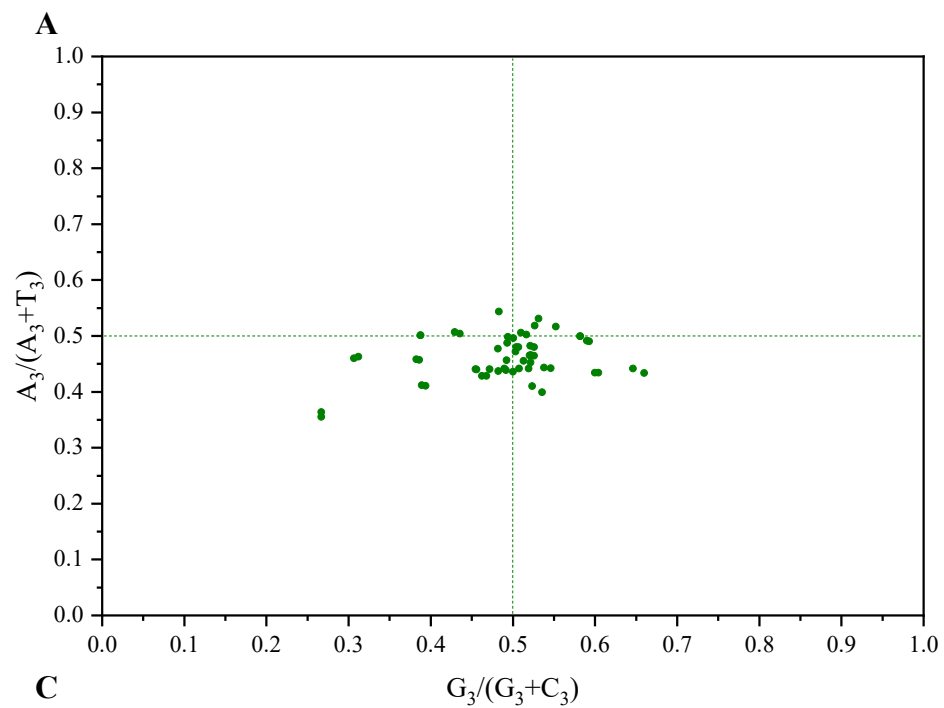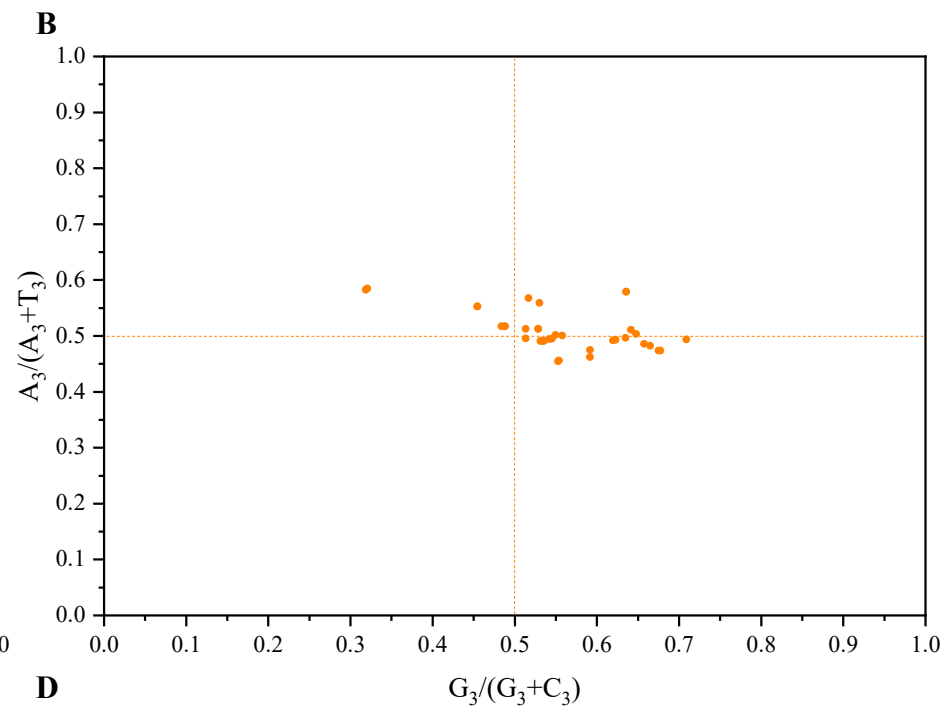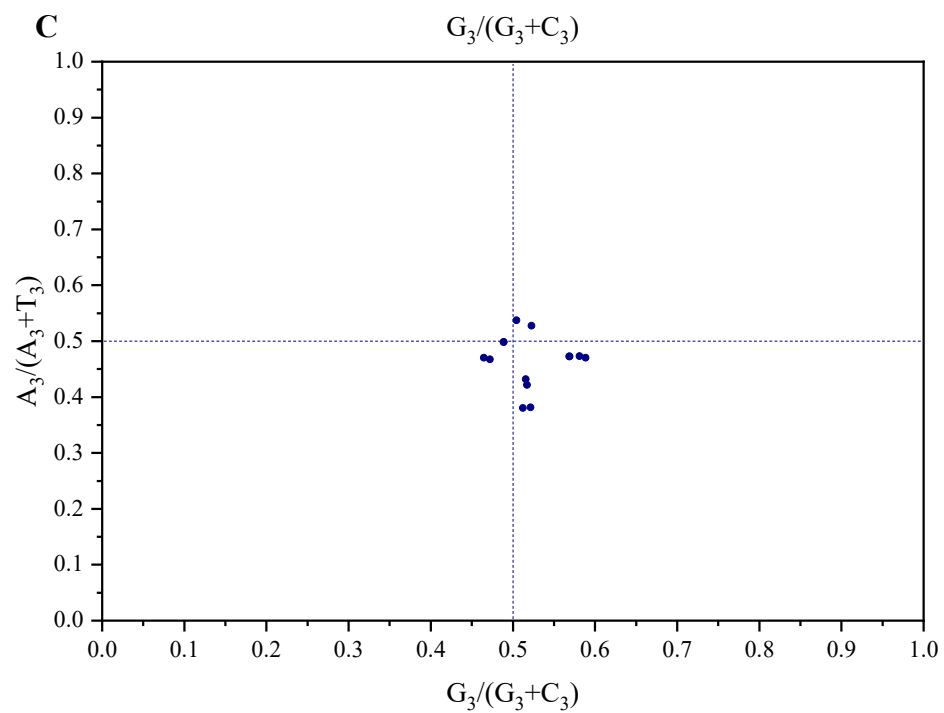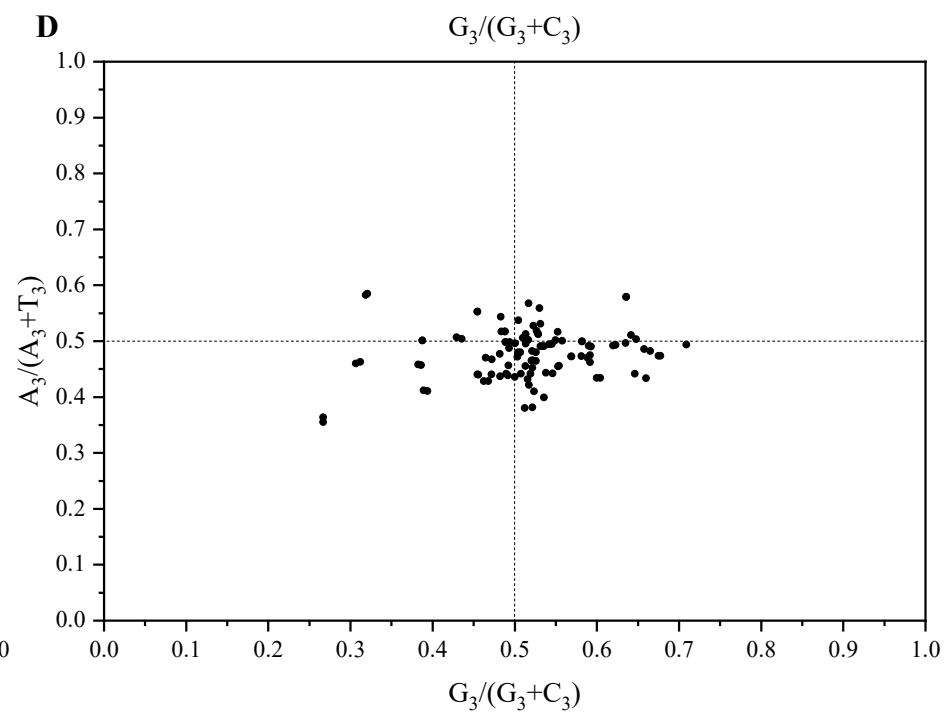

Supplement: Supplementary Figure 1 — The evolutionary relationship of four sisters genera (Roscoea, Cautleya, Pommereschea, and Rhynchanthus). [file DataSheet_1.zip › Supplementary Files/Figure S5.pdf]

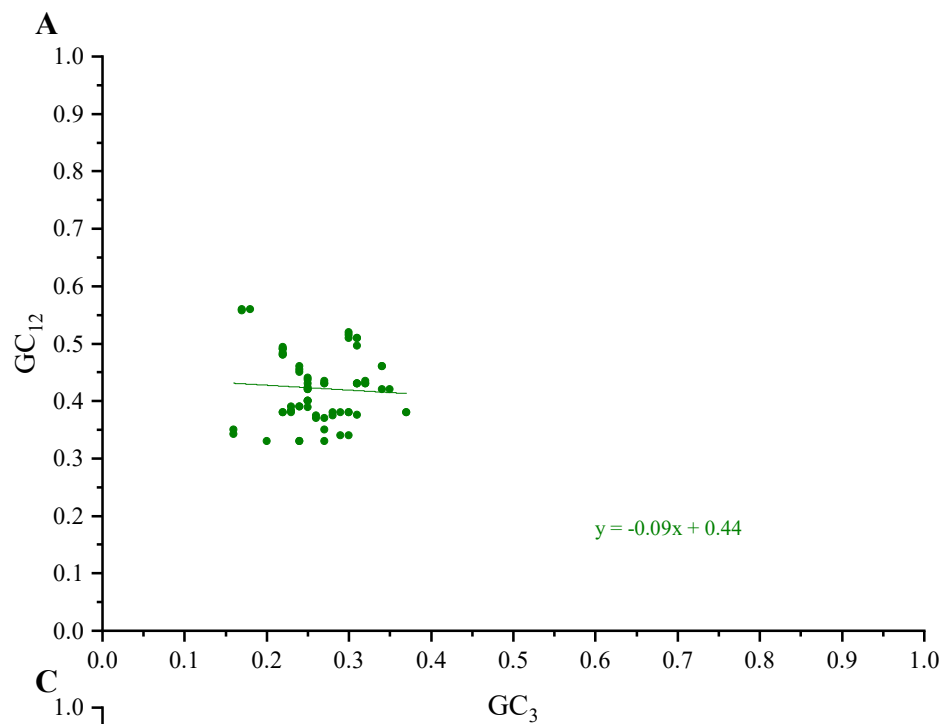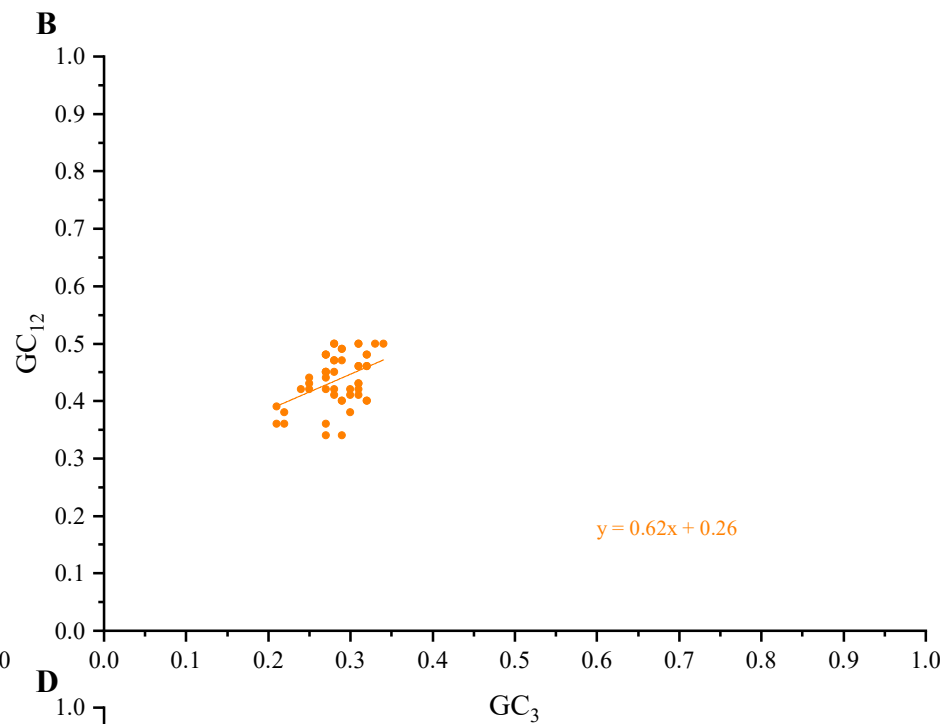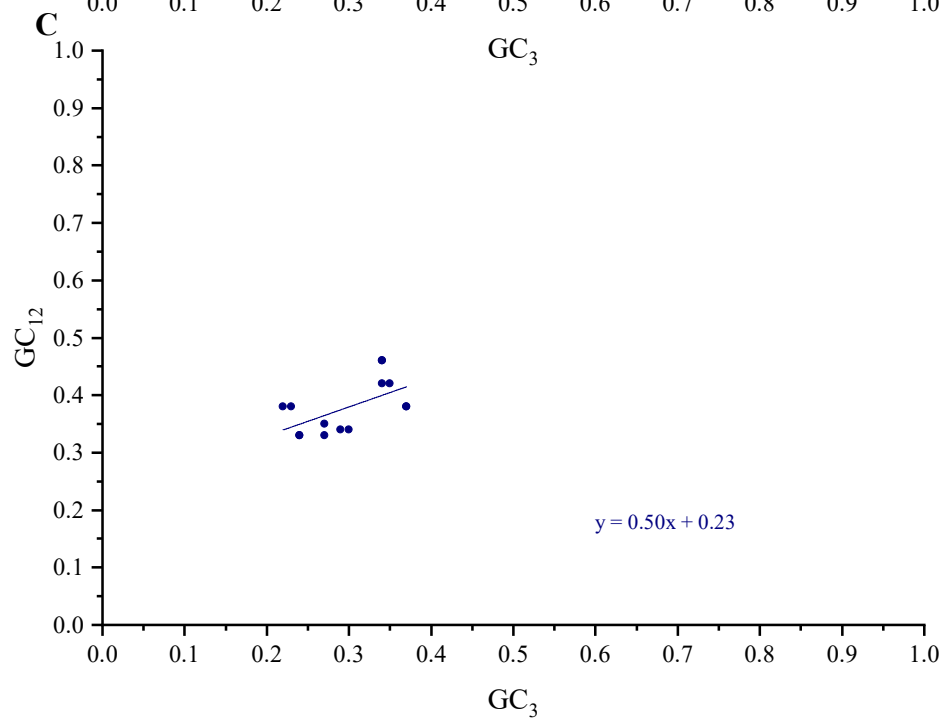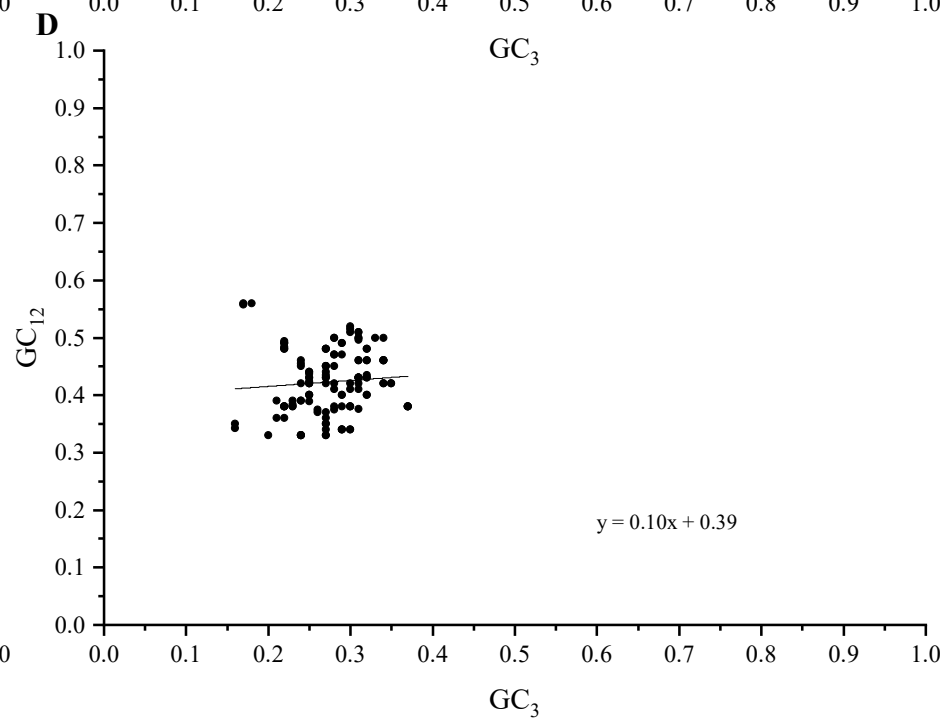

Supplement: Supplementary Figure 1 — The evolutionary relationship of four sisters genera (Roscoea, Cautleya, Pommereschea, and Rhynchanthus). [file DataSheet_1.zip › Supplementary Files/Figure S6.pdf]

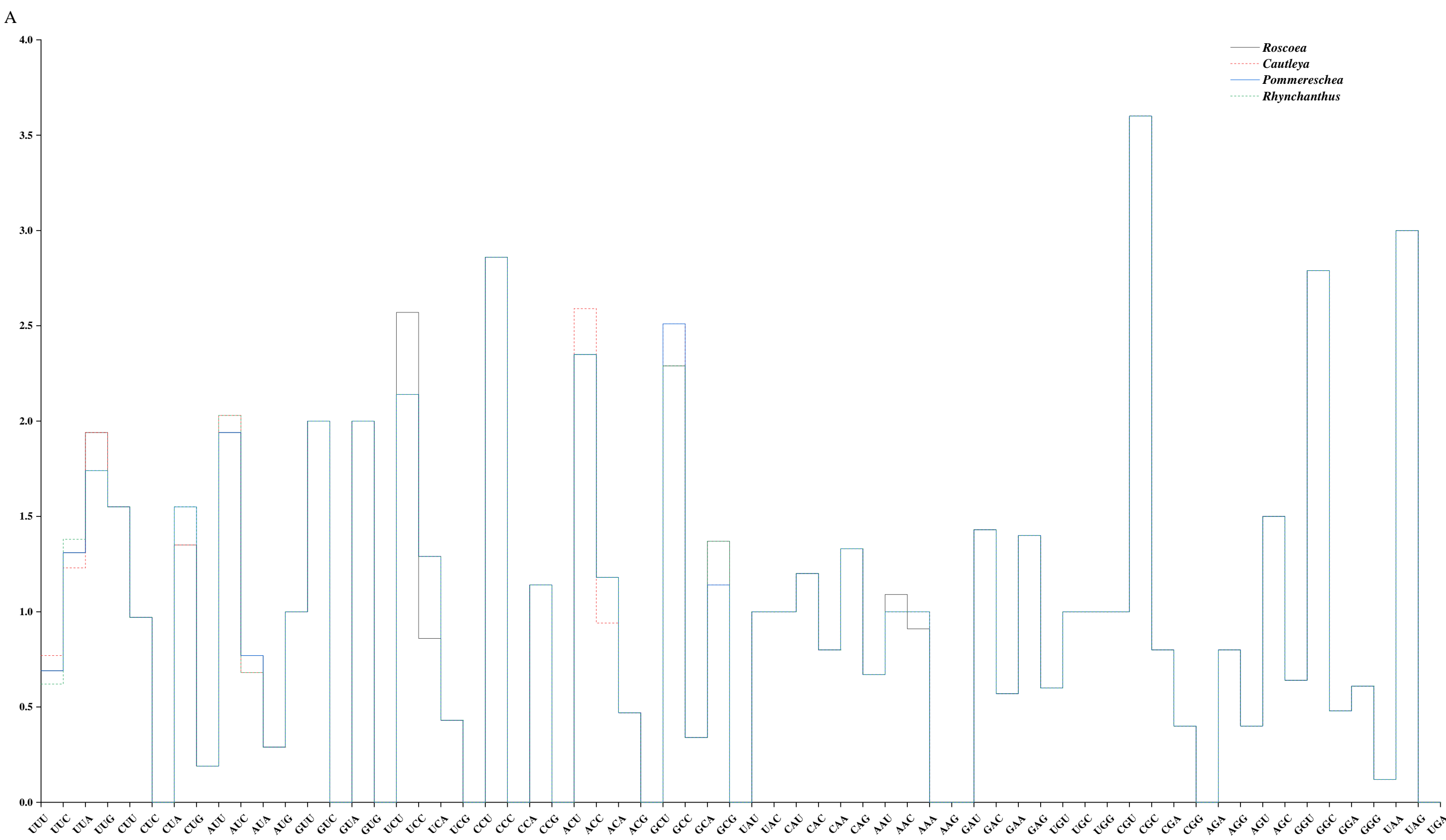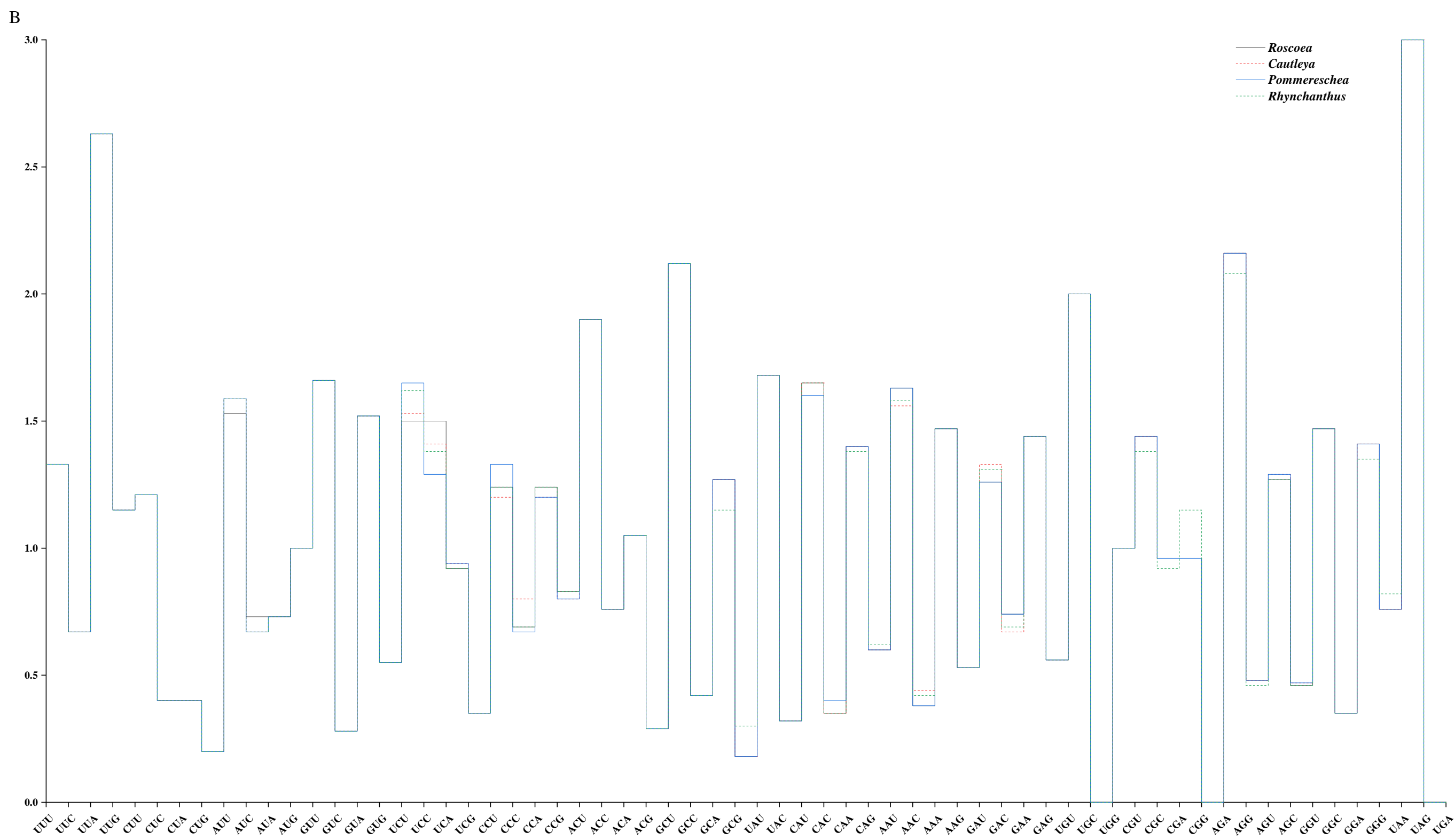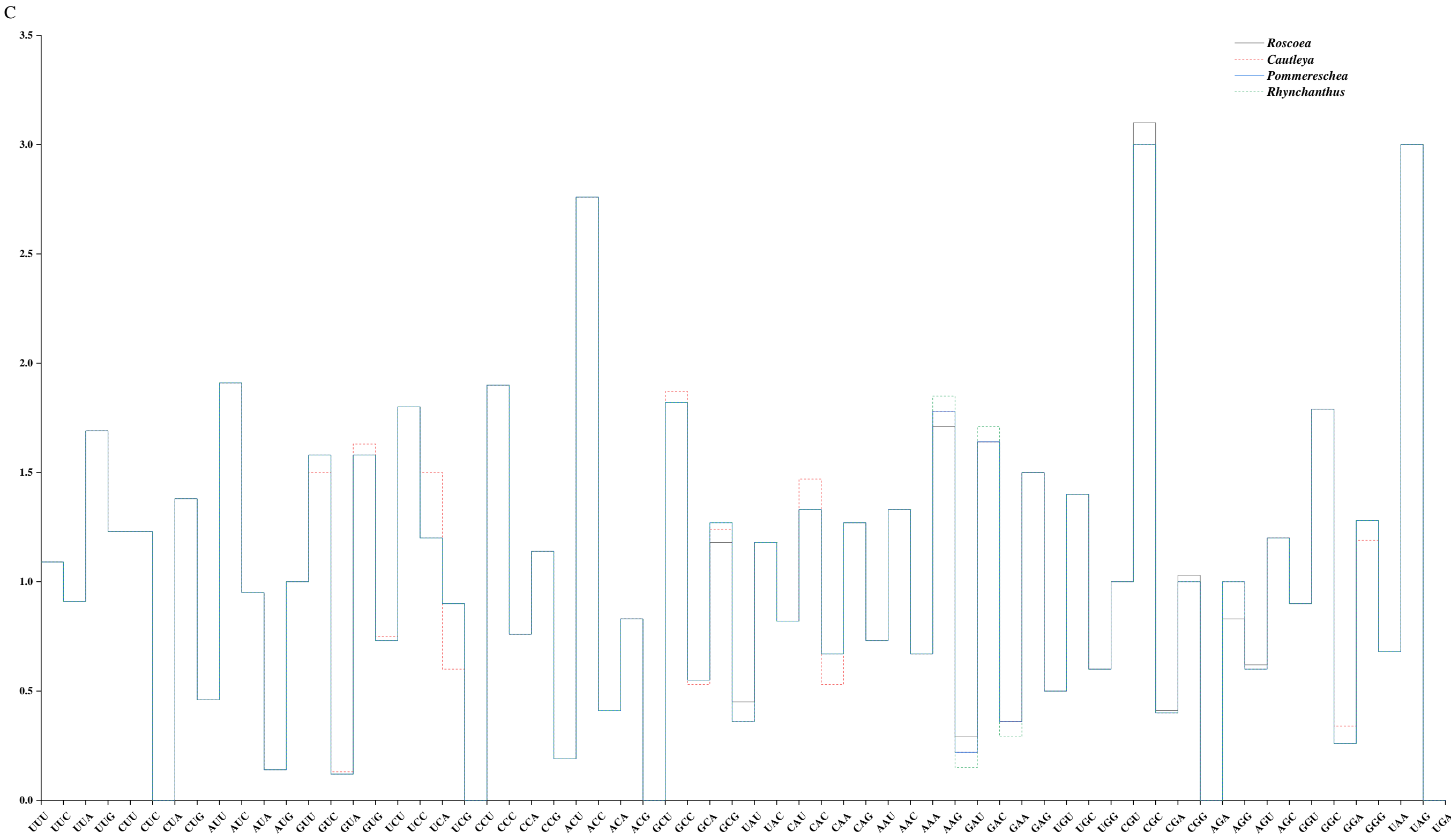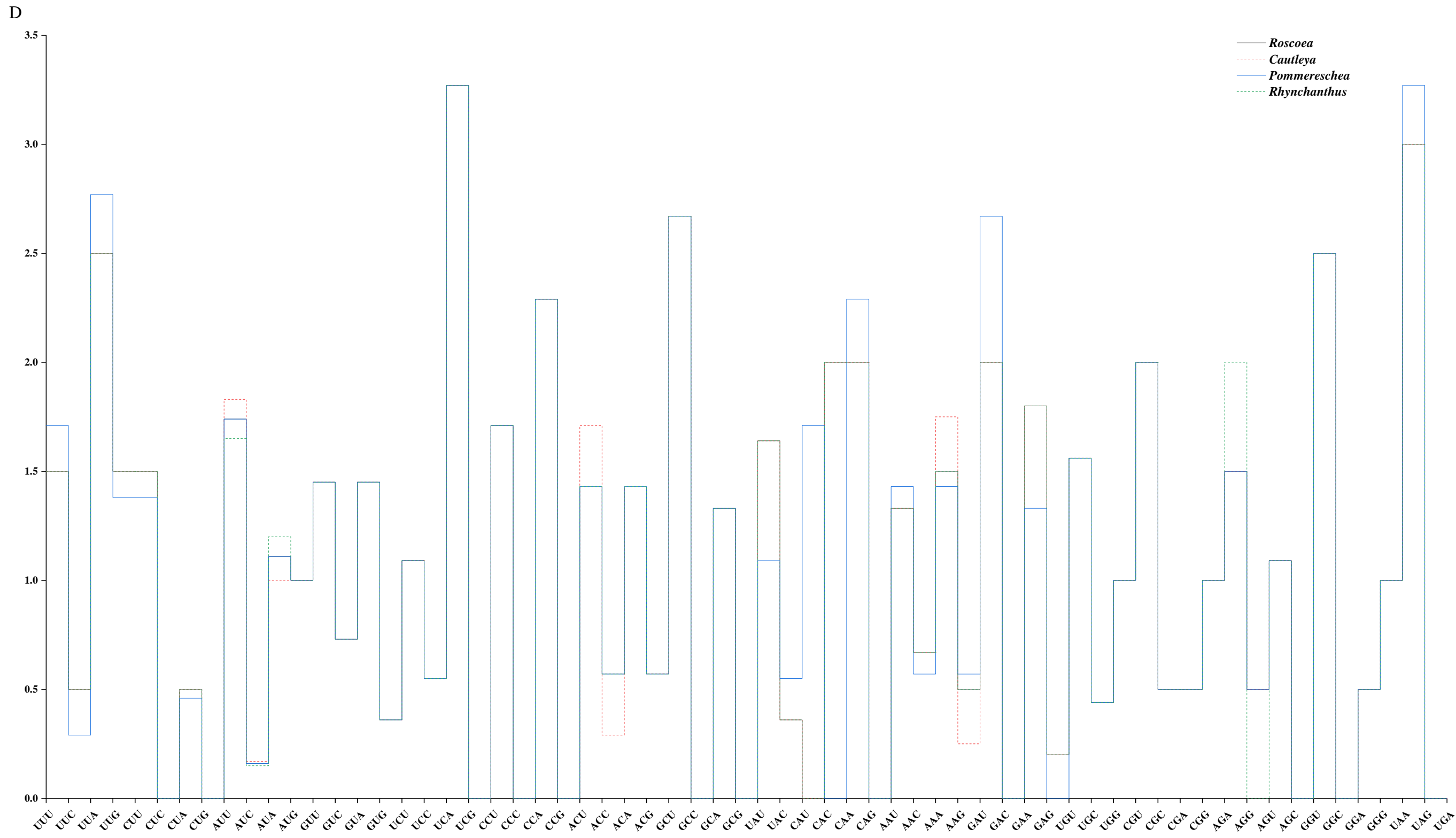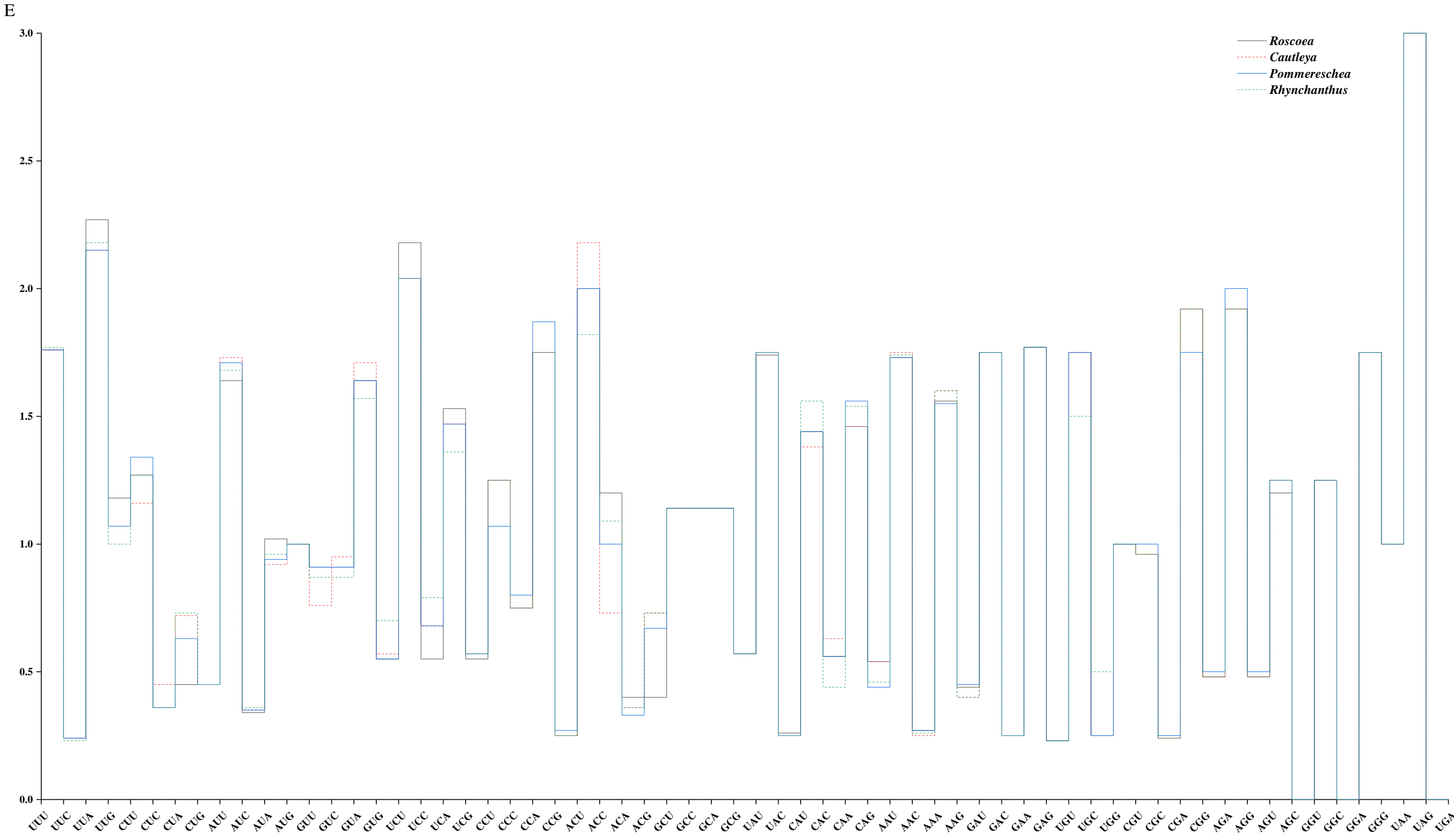

Supplement: Supplementary Figure 1 — The evolutionary relationship of four sisters genera (Roscoea, Cautleya, Pommereschea, and Rhynchanthus). [file DataSheet_1.zip › Supplementary Files/Figure S7.pdf]
